# What Drives Opposition to Social Rights for Immigrants? Clarifying the Role of Psychological Predispositions

**DOI:** 10.1177/00323217241228456

**Published:** 2024-02-12

**Authors:** Carlo M Knotz, Alyssa M Taylor, Mia K Gandenberger, Juliana Chueri

**Affiliations:** 1 Department of Media and Social Sciences, University of Stavanger, Stavanger, Norway; 2 Swiss Graduate School of Public Administration (IDHEAP), University of Lausanne, Lausanne, Switzerland; 3 Swiss Forum for Migration and Population Studies and NCCR – on the move, University of Neuchâtel, Neuchâtel, Switzerland; 4 Department of Political Science and Public Administration, Vrije Universiteit Amsterdam, Amsterdam, The Netherlands

**Keywords:** immigration, IAT, authoritarianism, ethnocentrism, social dominance orientation

## Abstract

Why do people oppose granting social rights to immigrants? Previous research indicates that psychological predispositions such as authoritarianism or ethnocentrism are strong drivers, but our understanding of their roles is still incomplete. This is in part because studies have not yet systematically tested different psychological variables against other, but also in part because some other potentially important predispositions such as implicit bias and social dominance orientation have so far been overlooked. We address this gap using original data from survey experiments conducted in six countries (Denmark, Germany, Sweden, Switzerland, the United Kingdom and the United States). We find consistent effects of ethnocentrism and social dominance orientation, a less robust effect of authoritarianism and no effect of implicit bias. In substantive terms, we find that a belief in ethnocentric stereotypes and a desire for dominance are the central factors driving opposition to immigrants’ social rights.

## Introduction

The inclusion of immigrants into their host countries’ social protection systems has been one of the most salient and contentious political issues of recent years, with one notable example being the debates in western Europe about the potential threat of ‘welfare tourism’ connected to the European Union’s (EU’s) 2004 eastern enlargement (Gago, 2021). Unsurprisingly, there has been a great deal of scholarly interest in the politics of immigrants’ social rights (see e.g. Careja and Harris, 2022 for a recent review), and a particular focus in this area of research has been on the determinants of individual-level attitudes towards the social rights of immigrants (e.g. Crepaz, 2020; Hooijer, 2021).^1^

One prominent explanation for why people oppose granting social rights to immigrants highlights the role of material self-interest and specifically that of (perceived) competition with immigrants for benefits and other fiscal resources (Ford, 2016; Hooijer, 2021). However, while being straightforward and intuitive, this explanation holds only under quite restrictive conditions (see Hooijer, 2021: 1383) and has, arguably as a result, a rather mixed empirical record (Careja and Harris, 2022: 125).

The second major approach, emphasising the role of psychological predispositions, has been more successful.^2^ Specifically, studies have found robust and strong effects of ethnocentrism (e.g. Ford, 2016; Hjorth, 2016), that is a predisposition to believe in ethnocentric stereotypes of foreigners as ‘inferior’ and to engage in ‘us versus them’ thinking (Kam and Kinder, 2012). Others found similar effects of authoritarianism (Crepaz, 2020), which is a deep-seated aversion to diversity and a desire for social order and conformity (Feldman, 2003).^3^

However, despite these crucial insights, previous research on these psychological predispositions still leaves important gaps. For one, existing studies have focused on the effects of single predispositions, but no study has, to our knowledge, directly compared the effects of different psychological variables in a single analysis. This is problematic because, although they are conceptually distinct, variables like ethnocentrism and authoritarianism tend to correlate empirically (e.g. Hooghe, 2008), and it is therefore unclear to what extent analyses that focused on single variables have really uncovered their true impacts.

The second (and larger) problem is that there are also other psychological predispositions that could plausibly drive attitudes towards immigrants’ social rights – but which have so far not been considered. One of these is social dominance orientation (SDO), which can be described as a brute and chauvinistic desire to live in a hierarchical society where one’s own group dominates over others (Pratto et al., 1994). SDO is in general a strong predictor of attitudes towards both perceived out-groups and towards ‘hierarchy-attenuating institutions’, such as social protection policies (Pratto et al., 1994: 743), meaning it can be expected to also affect attitudes towards the equal inclusion of immigrants into welfare states (see also Pratto et al., 1998). A second overlooked possibility is that opposition towards immigrants’ social rights is in part driven by subconscious or *implicit* attitudes and biases (Greenwald and Banaji, 1995). A large literature spanning psychology and political science has already linked implicit biases to different types of explicit attitudes and behaviours, including, for instance, voting and party preferences (e.g. Greenwald et al., 2009) but also explicit attitudes towards immigrants and towards benefit claimants (de Vries, 2017; Pérez, 2010). It is thus possible that explicitly held attitudes towards the social rights of immigrants could be influenced by underlying implicit biases.

The focus on ethnocentrism and authoritarianism in previous research also creates a final problem: a potentially skewed image of the socio-demographic basis of the opposition towards social rights for immigrants. Both authoritarianism and ethnocentrism are known to be strongly linked to low educational attainment (Dekker and Ester, 1987; Hooghe, 2008). Combined with the findings on the role of material self-interest (Hooijer, 2021), this creates an image of opposition to immigrants’ social rights as most pronounced among the lower educated and/or economically weak. This image is, however, difficult to reconcile with findings indicating that opposition towards the inclusion of immigrants into welfare states is actually widespread across different socio-demographic groups (e.g. Marx and Naumann, 2018). Implicit bias and SDO, which are more uniformly spread or, in the case of SDO may actually be linked to higher social status (Nosek et al., 2007; Pratto et al., 1994: 756), can provide complementary accounts.

Our contribution is to address these gaps and to clarify the effects of psychological predispositions on people’s attitudes towards immigrants’ social rights. We use original survey data collected in six countries (the United States, the United Kingdom, Germany, Denmark, Sweden and Switzerland) to test for and compare the effects of ethnocentrism, authoritarianism, SDO and implicit bias (the latter being measured with an Implicit Association Test (IAT); Greenwald et al., 1998) on attitudes towards immigrants’ social rights, which we measure indirectly with a vignette experiment (building on, e.g. Reeskens and van der Meer, 2017).

To preview our findings, we can on one hand confirm that ethnocentrism is indeed a strong and robust driver of opposition to immigrants’ social rights, and we also find a similar (and independent) effect of SDO. On the other hand, we find that authoritarianism has more limited effects, which appear only in the case of immigrants with stereotypically darker skin tone and those from non-Western countries. We find no effect of implicit bias.

In substantive terms, our results indicate that opposition to immigrants’ social rights is driven in part by a predisposition to believe in ethnocentric stereotypes and in part by a desire for a hierarchically organised society in which the stronger dominate the weak. The effect of authoritarianism, that is an aversion to social diversity, is focused on immigrants that are culturally or racially different from the mainstream population. Indirectly, our findings also qualify the image of opposition towards immigrants’ social rights as a defensive response by those in economically or culturally vulnerable positions (e.g. Crepaz, 2020; Hooijer, 2021) and indicate that it originates at least in part from a position of strength and a desire to maintain that position (adding to related research on the ‘populism of the privileged’; see De Cleen and Ruiz Casado, 2023).

The remainder of this article proceeds as follows: The following section discusses relevant psychological explanations for opposition towards immigrants’ social rights, specifically the two that were considered in previous research and the two candidate explanations we propose. The third section introduces our survey and the experiments, while the fourth section presents our results. The final section concludes.

## Opposition to Immigrants’ Social Rights: The Role of Psychological Predispositions

### Existing Accounts: Ethnocentrism and Authoritarianism

Next to material self-interest and concerns about benefit competition (Hooijer, 2021), ethnocentrism and authoritarianism have been identified in previous research as important drivers of opposition towards immigrants’ social rights. Starting with the former, ethnocentrism is a general predisposition to engage in ‘us versus them’ thinking or, put differently, to perceive the world as divided into one’s own in-group on the one side and all outsiders on the other (Kam and Kinder, 2007, 2012). The word ‘general’ is key here: Persons with an ethnocentric predisposition apply a group-centric perspective not just to specific social groups but as a way to make sense of the world and of group relations in general.

This ‘us versus them’ worldview goes along with stereotypical thinking, or a belief that groups can generally be characterised by specific attributes or behaviours. Certain attributes or ‘typical’ behaviours are then ascribed to one’s own in-group, and others to relevant out-groups. Importantly, attributes or behaviours that are seen as typical for one’s in-group are seen as superior while out-groups who differ (or are perceived to differ) from the in-group in their attributes or behaviours are seen as generally inferior. In other words, from an ethnocentric perspective, the in-group and its stereotypical attributes are the standard against which other groups are measured – and groups that are perceived to differ from the ‘superior’ standard set by the in-group are accordingly devalued. Perceptions of one’s in-group as superior to others are of course a common human trait (Tajfel et al., 1971), but people with an ethnocentric predisposition engage in such thinking to a greater degree than others. For example, ethnocentrism manifests itself empirically in a clearly stronger inclination to see one’s own nation or ethnic group as ‘hard-working’, ‘honest’ and ‘intelligent’ and others as ‘lazy’, ‘untrustworthy’ and ‘unintelligent’ (Kam and Kinder, 2012).

While it is not difficult to see how a belief in such negative stereotypes would lead to differential treatment and discrimination against others across the board, a specific and direct link to opposition to immigrants’ social right is provided by the theory of deservingness heuristics (Petersen, 2015: 65), which predicts that people are generally unwilling to share with or support those they see as lazy or as having ulterior motives.

A second psychological variable that has been proposed to explain attitudes towards immigrants’ social rights is authoritarianism (Crepaz, 2020).^4^ Authoritarianism is defined as a predisposition to seek conformity, obedience to rules and stability – or, conversely, an aversion to diversity and disorder (Feldman, 2003). As Feldman (2003) argued, people with an authoritarian predisposition have a generally pessimistic view of others and their intentions, and accordingly fear that allowing or even promoting greater individual liberty will only produce social disorder and instability. Their preference is thus to limit individual freedom and to enforce social order and conformity. Crucially, this desire for order, control and conformity also leads authoritarians to be highly sceptical of the potential for very different lifestyles, religious orientations or norms to peacefully coexist. This in turn leads to opposition towards immigration, an obvious driver of societal diversity, and of political measures that would encourage or sustain immigration such as granting immigrants social rights (Crepaz, 2020). While authoritarianism is identical to ethnocentrism in its expected effect – increased opposition to social rights for immigrants – the underlying mechanisms are distinct: In the case of authoritarianism, opposition to immigrants’ social rights is driven by an aversion to diversity and a desire for order and conformity rather than a belief in stereotypes.

### Candidate Predictors: Dominance Orientation and Implicit Bias

While ethnocentrism and authoritarianism are highly plausible predictors of opposition towards social rights for immigrants, psychological research has also identified other predispositions that can, in theory, be expected to have similar effects.

The first of these is SDO. As Pratto et al. (1994) have originally defined it, SDO is essentially a desire to live in a hierarchical society in which one’s own group dominates over other, weaker groups – or, conversely, an aversion to an egalitarian society in which multiple groups of equal status coexist. While SDO might seem to simply capture political ideology, research has shown that SDO is really a more fundamental psychological predisposition, similar to ethnocentrism and authoritarianism (Pratto et al., 1998).

Already Pratto et al.’s (1994: 743) seminal article predicted an association between SDO and increased opposition towards policies that reduce inequality between native citizens and immigrants, and it is indeed not difficult to see how giving immigrants (more) equal access to social protection directly militates against a strong dominance orientation: For one, to the extent that immigrants are economically disadvantaged, giving them equal access to social protection would work to reduce these disadvantages and reduce overall social inequality. Second, the mere fact that immigrants are treated as equals when it comes to access to social protection is inherently inconsistent with the idea of a hierarchical society.

In addition, social psychologists have produced considerable evidence that SDO reduces support for policies that reduce hierarchies and redistribute resources towards weaker groups, notably social welfare and social insurance policies (e.g. Jedinger and Kaminski, 2023; Pratto et al., 1998). Relatedly, research has also found strong links between SDO and anti-immigrant attitudes (e.g. Thomsen et al., 2008). In light of these findings, it is plausible that SDO would also drive opposition to granting immigrants social rights more specifically.

SDO is conceptually distinct from both authoritarianism and ethnocentrism. While SDO consists of a personal desire for domination over others, authoritarianism by contrast is an inclination to submit to a larger authority that enforces social order and obedience. This also means that authoritarians and persons high in SDO are likely to be relatively distinct groups (Pratto et al., 1994: 744). Similarly, SDO is not synonymous with ethnocentrism. On one hand, SDO can be expected to correlate with ethnocentrism, since ethnocentric stereotypes provide a form of ‘legitimising myth’ that justifies the dominance of an in-group over others (Pratto et al., 1994: 742). Nevertheless, the two variables still capture substantively different concepts: SDO is a desire for dominance, while ethnocentrism is a belief or a worldview that can be used to justify that desire.

The three predispositions discussed so far all operate within people’s awareness in the sense that people can be asked more or less directly about their views about diversity, intergroup relations or authority, and will then provide answers that reflect their underlying ethnocentrism, authoritarianism or dominance orientation. However, a large amount of research in psychology and other fields has shown that people also have ‘implicit’ attitudes and biases that lie beneath conscious awareness, and that these implicit attitudes can in turn influence explicit attitudes and behaviour in subtle and often unrecognised ways (Banaji, 2001; Greenwald and Banaji, 1995; Nosek et al., 2007).^5^

Implicit attitudes and biases are a form of rudimentary, automatic and usually unconscious emotional responses to a given object or person as simply ‘bad’ or ‘good’, ‘pleasant’ or ‘unpleasant’, and so on (Pérez, 2013: 277). Such implicit attitudes are thought to be created by long-term exposure to a specific cultural and political environment and its taken-for-granted beliefs, in the process of which certain associations become so deeply ingrained in someone’s memory that they become automatic and are no longer consciously recognised. Implicit negative attitudes towards immigrants, for example, could be the result of exposure to discourses that present immigrants in a negative light and which over time create unconscious emotional associations between ‘immigrant’ and ‘bad’ or ‘unpleasant’ (Banaji, 2001; Greenwald and Banaji, 1995).

Implicit biases have been shown to influence people’s explicit political and social attitudes and their behaviour towards perceived out-group members. For example, Pérez (2010) found that US Americans have implicit associations of ‘Latino immigrant’ with negative words (i.e. ‘bad’) and ‘White immigrant’ with positive words (i.e. ‘good’), and he found that these implicit biases significantly predict people’s immigration policy attitudes. Studies have also found that implicit attitudes exist when it comes to social protection policies and claimants (de Vries, 2017; Jensen and Petersen, 2017) and, more generally, that negative implicit attitudes towards a group can affect people’s willingness to engage in economic interactions with that group (Stanley et al., 2011). The fact that implicit biases exist and that such biases can influence attitudes towards resource sharing and public policies creates a possible link between implicit bias and attitudes towards immigrants’ social rights.

Although implicit attitudes and biases are often correlated with corresponding overt or ‘explicit’ attitudes, implicit and explicit attitudes are distinct phenomena (Nosek et al., 2007; Nosek and Smyth, 2007). In other words, someone can overtly express tolerance towards some group while at the same time still holding implicit biases against them.

To sum up this theoretical discussion, we formulate the following set of (not mutually exclusive) hypotheses about the drivers of opposition to social rights for immigrants:

H1: Persons higher in ethnocentrism are more opposed to social rights for immigrants.
H2: Persons higher in authoritarianism are more opposed to social rights for immigrants.
H3: Persons higher in SDO are more opposed to social rights for immigrants.
H4: Persons higher in implicit bias are more opposed to social rights for immigrants.

## Survey, Measurement and Model Specification

### Survey

To test whether and how strongly the four psychological predispositions are related to opposition to social rights for immigrants, we use data from an original public opinion survey (Gandenberger et al., 2021) that was conducted in six countries (Denmark, Germany, Sweden, Switzerland, the United Kingdom and the United States).^6^ The survey fieldwork was conducted in the summer and fall of 2021 using samples of around 3000 respondents in the US and Germany, and of around 1500 respondents in the other countries.^7^ These six countries were chosen because they differ in central macro-level variables such as the design of welfare state institutions (Esping-Andersen, 1990) and immigration policies and histories (Koopmans and Michalowski, 2017) that have been shown to affect both welfare state attitudes in general and attitudes towards the social rights of immigrants specifically (Larsen, 2008, 2020). By drawing on data from this diverse set of countries, our study improves over existing single-country studies (e.g. Ford, 2016; Reeskens and van der Meer, 2019).

Respondents were recruited from online panels operated by a European market research and opinion polling company (*Bilendi*) using quotas for age, gender, education and geographic area (urban vs rural).^8^ This recruitment method is increasingly widely used and, although it does obviously not achieve the same level of representativeness as traditional probability sampling, recent systematic evaluations have shown that effect estimates from experiments based on such data can be generalised to the underlying populations (e.g. Coppock et al., 2018).

### Measurement: Experiments and Instruments

**Figure 1.**
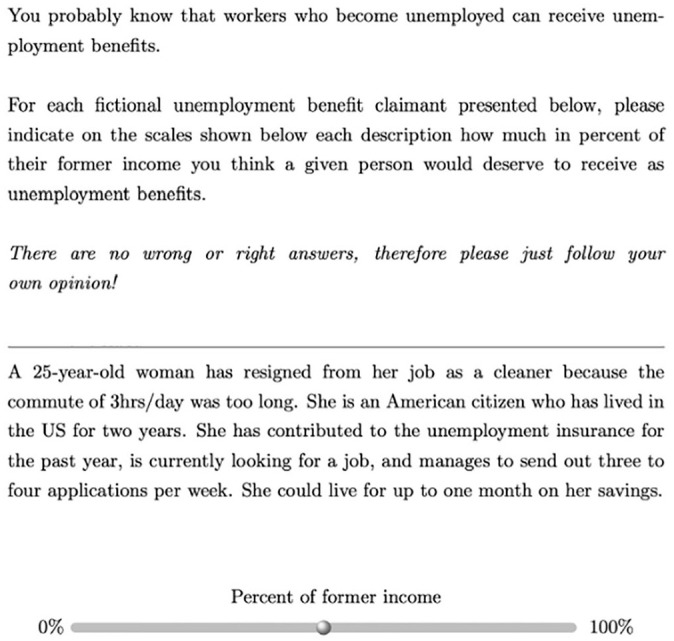
Example Vignette (US Version).

We measure our dependent variable, respondents’ attitudes towards social rights for immigrants, indirectly using a fractional factorial vignette experiment (see e.g. Jasso, 2006). In our experiment, respondents were shown brief vignettes of fictional unemployed persons which varied randomly in a set of predefined attributes (including notably their immigration background; see also below).^9^ Respondents were then asked to indicate on a continuous 0–100 scale what percentage of their previous income each unemployed person should receive in the form of benefits (see Figure 1 for an example vignette) – that is respondents were asked to rate the ‘deservingness’ of each vignette to benefits (van Oorschot, 2000).^10^ Opposition to social rights for immigrants should manifest itself in lower benefits being granted to vignettes featuring immigrants compared with vignettes featuring native citizens, all else being equal (see also Reeskens and van der Meer, 2019 for a more detailed discussion of the link between deservingness perceptions and anti-immigrant bias).

By measuring opposition to social rights of immigrants indirectly, via what is essentially an experiment on welfare deservingness perceptions (van Oorschot, 2000), we follow the examples of several previous studies that followed the same approach (e.g. Ford, 2016; Kootstra, 2016; Reeskens and van der Meer, 2019). This approach has several advantages: For one, the random composition of vignettes makes it possible to isolate the effect of having an immigrant background from potential confounders, specifically variables that are often correlated with immigration background (e.g. employment record) and which can affect deservingness perceptions and social policy attitudes (Reeskens and van der Meer, 2019; see also Hainmueller et al., 2014; Jasso, 2006). In addition, vignette experiments reduce the problem of social desirability bias, which is particularly important in the present case, since the social rights of immigrants have been shown to be a sensitive topic where respondents often do not disclose their true attitudes when asked directly (Cappelen and Midtbø, 2016). Social desirability bias is reduced in vignette experiments because respondents are not directly asked about their attitudes towards immigrants and their rights and – because vignettes vary in several attributes other than immigration background – respondents may not even notice the true purpose of the experiment (Horiuchi et al., 2022). Finally, despite their obviously artificial nature, vignette experiments have been shown to have external validity (Hainmueller et al., 2015). Our experiment focuses on unemployment benefits, since these are relatively similarly structured in different countries (Clasen and Clegg, 2011: 4), which means that respondents’ evaluations are less likely to be influenced by differences in countries’ benefit programmes.

Table 1 lists all vignette attributes and their respective levels. The central attribute for the purposes of our analysis is the fictional person’s citizenship. We selected nationalities that would form a rough ethnic hierarchy (Hagendoorn, 1995), spanning from native citizens over culturally, ethically and linguistically close neighbouring countries to (relatively) distant Ukrainians and even more distant Nigerians and Afghans.^11^ The other attributes featured on the vignette were selected based on the findings from deservingness research, according to which people see others as more deserving the less control they have over their situation; the more effort they make to help themselves; the more they have contributed to society in the past; the more they are in need; and the more similar they are in their social identity (Petersen, 2012; van Oorschot et al., 2017). We also added some further attributes, namely gender, age, residency and occupation to the vignettes, which helps us to further verify that any effects of a foreign nationality are not driven by respondents’ potential assumptions about the socio-demographics of different immigrant groups (Herda, 2010). The distributions of vignette evaluations in the different countries (provided in the Online Appendix; see Figure S-14) are relatively symmetric and show no tendencies towards extremely high or low evaluations.

**Table 1.** Vignette Attributes and Levels.

Attribute	Levels
Reason for unemployment	Resigned because work was contradictory to religious values
	Resigned because a 3-hour commute was too long
	Resigned because of excessive overtime
	Lost job due to COVID-19 pandemic
Time during which taxes and contributions were paid before unemployment	Past year, past 2 years, past 4 years and past 8 years
Efforts to find new work	Not currently looking
	1–2 job applications per week
	3–4 job applications per week
	5–6 job applications per week
Occupation	Cleaner
	Lab technician
	Food engineer
	Accountant
Time able to live on savings	Up to 1 month
	Up to 3 months
	Up to 6 months
	Up to 1 year
Gender	Male, female
Age	20, 40, 55 years
Citizenship	German/American/British/Swedish/Swiss/Danish
	Austrian/Canadian/Irish/Norwegian/German/Swedish
	Ukrainian
	Afghan
	Nigerian
Residency in country	Born in country
	10 years
	5 years
	2 years

To measure our core independent variables, implicit bias, ethnocentrism, authoritarianism and SDO, we relied on established methods and instruments. To measure authoritarianism, we used a battery of child-rearing items in which respondents are asked which of the following qualities they would like children to be taught at home: good manners, obedience, religious faith, independence, imagination, and tolerance and respect for others. The first three options reflect an authoritarian mindset, whereas the other three are related to anti-authoritarianism. We then constructed an indicator of authoritarianism as the difference between the number of authoritarian and anti-authoritarian qualities selected by a given respondent (building on Tillman, 2013; see also Crepaz, 2020; Feldman, 2003). The resulting indicator ranges from −3 (very anti-authoritarian/libertarian) to 3 (very authoritarian) and, corresponding to previous findings (Crepaz, 2020: 1258), is roughly normally distributed in the different countries (see Figure S-9 in the Online Appendix).

To measure ethnocentrism, we followed the approach by Kam and Kinder (2012) and created three item batteries in which respondents were asked to rate different groups (the native-born, immigrants from other North American/European countries, immigrants from post-Soviet countries, immigrants from the Middle East, immigrants from Africa) on three dimensions: to what extent they are ‘hard-working’ or ‘lazy’, ‘intelligent’ or ‘unintelligent’, and ‘trustworthy’ or ‘untrustworthy’. Respondents rated each group on each dimension on 1–7 scales. We then constructed an indicator of ethnocentrism as the average difference between the rankings given to the native-born and immigrant groups.^12^ Note that we calculate this indicator only for the majority of respondents who are themselves native-born and not for those with an immigration background, the reason being that matching respondent backgrounds to the groups covered by our ethnocentrism indicator is not straightforward and would in part be based on relatively small groups of respondents from specific backgrounds. A score of 0 indicates that a given respondent sees immigrants as no less hard-working, intelligent or trustworthy than the native-born (i.e. no ethnocentrism). Positive scores indicate a more favourable view of the native-born compared with immigrants, while negative scores indicate a more positive view of immigrants compared with the native-born. Corresponding to findings in earlier studies, our samples lean towards in-group favouritism (cf. Kam and Kinder, 2012: 328), with the exception of the US, where no trend to either side is visible (see Figure S-10 in the Online Appendix).

SDO was measured with the short 4-item scale developed by Pratto et al. (2013). Respondents were asked to what extent they agree with the following four statements: ‘In setting priorities, we must consider all groups’, ‘We should not push for group equality’, ‘Group equality should be our ideal’ and ‘Superior groups should dominate inferior groups’, and could indicate their position on a scale from 0 (‘Oppose extremely’) to 10 (‘Favour extremely’). The overall SDO indicator was calculated as the scale mean, where higher scores indicate greater SDO.^13^ Our samples are mostly low to intermediate in SDO, and we are able to replicate the significant difference between men and women that was previously found by Pratto et al. (1994: 747).^14^

To measure implicit bias, we used an IAT (see Greenwald et al., 1998). The IAT is a widely used and extensively validated method to measure implicit attitudes and biases and functions (very simply put) by measuring the speed with which respondents can make certain associations compared with others.^15^ Note that we fielded the IAT only in Germany and the US, and with only half of each sample (randomly selected).^16^

We use a specific version of the IAT, namely the skin-tone IAT, which is designed to measure respondents’ implicit biases against darker- compared with lighter-skinned persons. The skin-tone IAT is set up as follows: Respondents are shown either an image of a light- or dark-skinned person or a pleasant or unpleasant word (e.g. ‘Glorious’, ‘Beautiful’, ‘Dirty’, or ‘Rotten’; see Table S-1 in the Online Appendix for the exact word stimuli) in the centre of their screen.^17^ Respondents then use their keyboard to sort this stimulus into categories that are shown in the upper corners of the screen. In some parts of the test, ‘compatible’ categories (dark-skinned and ‘unpleasant’ or light-skinned and ‘pleasant’) are shown on the same side of the screen, while respondents see ‘incompatible’ pairings (light-skinned and ‘unpleasant’) in other parts. The core idea behind the test is that respondents should be able to sort targets more quickly when these are presented in a ‘compatible’ setting and they use the same key for categories that are implicitly associated (e.g. dark-skinned and ‘unpleasant’). The differences in response times (*latencies*) between ‘compatible’ and ‘incompatible’ pairings were then translated into *D*-scores, an individual-level measurement of implicit bias.^18^ Positive *D*-scores indicate a tendency to associate dark-skinned with ‘unpleasant’ and light-skinned with ‘pleasant’.

We chose the skin-tone IAT for two reasons: The first reason is that this IAT and its stimuli have been thoroughly validated and are known to work across different political and linguistic contexts (Nosek et al., 2007), which is crucial in our case. As shown in the Online Appendix (Figures S-7 and S-8), our *D-*scores closely resemble those found in previous research, and we can also successfully replicate the positive relationship between implicit bias and age found by Nosek et al. (2007: 32). Second, although skin tone is of course not the same as immigration status, the two are correlated in our experimental set-up in that two of the immigrant groups we use in the vignette experiment differ in their (stereotypical) skin tones from the majority populations in our six countries. Thus, if people hold biases against darker-skinned persons and if that bias leads them to discriminate when it comes to social protection, then this should show up in our data in the form of significantly lower evaluations of at least those immigrants with a (stereotypical) dark skin tone.^19^

The four psychological variables are overall not strongly correlated with each other (see Tables S-2 through S-7 in the Online Appendix). The highest correlation we found here amounts to 0.32 (between authoritarianism and SDO in Denmark), with three other variable pairs achieving correlations of around 0.28, but most being significantly lower at around 0.10. This indicates that the four variables really measure distinct preconditions and personality attributes. The weak associations that do emerge are plausible. The IAT *D*-scores are weakly but statistically significantly associated only with ethnocentrism, which makes sense since both variables measure forms of bias and prejudice (Nosek et al., 2007). Ethnocentrism, SDO and authoritarianism are all positively and statistically significantly associated with each other (which is again in line with earlier findings; e.g. Pratto et al., 1994) but, again, the associations are substantively weak.

We control for a range of respondent demographics, including age (in years), educational attainment (below secondary; secondary; tertiary), gender (male; female), immigrant background (born in country vs not), labour market status (unemployed/employed) and economic resources (using an item that asks respondents how long they could sustain themselves on their savings if they lost their jobs: 2 weeks; 1 month; 3 months; 6 months; 1 year, or more than 1 year; entered as a linear covariate).^20^ In analyses that focus on the US data, we also control for race/ethnicity (White; Black/African American; Asian/Pacific Islander; Hispanic; Other).

We checked if the four psychological variables are correlated with socio-demographic variables (the results are reported in Figure S-13 in the Appendix). We find that ethnocentrism, authoritarianism and SDO are all to similar degrees and significantly negatively associated with education and positively with age. However, SDO stands out in that it is positively associated with greater socio-economic resources (greater savings, not being unemployed), which is not true for ethnocentrism or authoritarianism (ethnocentrism positively linked to unemployment, while authoritarianism is not associated with unemployment or financial resources).^21^ Implicit bias is only positively associated with age and, in the US, negatively with identifying as Black or African American, confirming the otherwise cross-cutting nature of implicit bias.

### Model Specification

Our data are hierarchical in nature – each respondent rated multiple vignettes and their ratings are then pooled in a common dataset – and contain both vignette-specific and respondent-specific (e.g. IAT *D*-score, ethnocentrism, etc.) variables. We therefore estimate linear multilevel (mixed-effects) regression models, in which the vignette evaluations given by respondents are the dependent variable. The models include dummies for all vignette attributes plus the mentioned respondent-level controls. Models that are run on the pooled data from all countries also include country-fixed effects.

Our central specification is a cross-level interactive model, in which we interact our core independent variables with a dummy that indicates if a given vignette profile has a foreign background or not. We use a binary dummy instead of the individual citizenship-categories since this allows us to also include a random effects term for this variable into the specification, as is required when models include a cross-level interaction (Heisig and Schaeffer, 2019).^22^

We use three dummy variables to capture different types of ‘foreignness’. The first is a simple native citizen versus immigrant (neighbouring/Ukrainian/Afghan/Nigerian) dummy. Two alternative dummies account for the potential relevance of perceived ethnic and racial hierarchies (Kootstra, 2016), that is people discriminate less against some immigrant groups than against others. The first of these alternative dummies captures the effect of skin-tone and contrasts groups with (stereotypically) light skin tone (native citizens, immigrants from neighbouring countries and Ukrainians) to those with (stereotypically) darker skin tone (Afghans and Nigerians). The second alternative dummy focuses more on cultural or ethnic differences and distinguishes between persons from WEIRD (Western, comparatively educated, industrialised, rich democracies; Henrich et al., 2010) countries (native citizens and those from neighbouring countries) and those from non-WEIRD countries (Ukrainians, Afghans and Nigerians).

We use the results of our interactive models to calculate the average marginal effects of a given dummy over values of our core independent variables (Brambor et al., 2006; Leeper et al., 2018). To reiterate, our main expectation is to find that higher values of authoritarianism, SDO, ethnocentrism and implicit bias are associated with a stronger negative effect of being an immigrant (or non-WEIRD/darker-skinned, respectively) on vignette evaluations.

## Results

### Baseline Models

**Figure 2.**
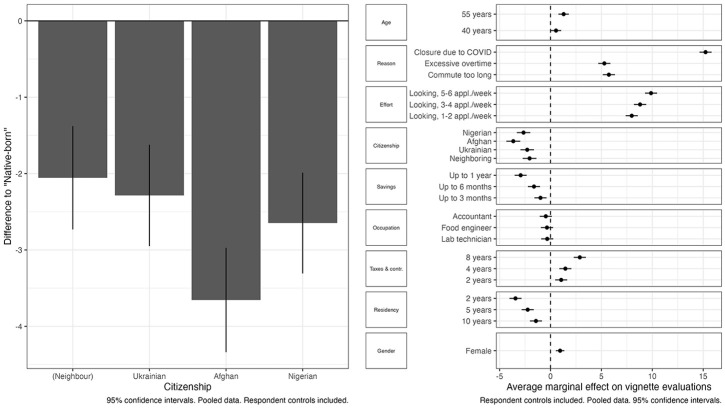
The Effects of Vignette Characteristics on Respondents’ Evaluations.

We start with the results of our pooled analysis in which we focus on the three independent variables that are available for all countries: ethnocentrism, SDO and authoritarianism (i.e. we focus on H1–H3). First, we estimate a baseline model in which we regress the vignette evaluation scores on all vignette variables (including the citizenship-attribute in its original, disaggregated form), all respondent controls and a set of country-fixed effects. Figure 2 displays the effects of the vignette attributes.^23^ The left-hand side graph shows our main pattern of interest, the bias against foreign citizens, while the right-hand side figure shows this pattern in the context of the effects of all vignette attributes.

As is immediately apparent from the left graph in Figure 2, foreign citizens are generally given lower evaluations – that is are seen as less deserving of unemployment benefits – than native citizens (the baseline category), all other factors held constant. The graph also indicates that the effects of the different citizenship categories are not generally different from each other, and this is confirmed when we test if the coefficients are statistically different. The only significant differences we find are those between Afghans and the other foreign nationalities.^24^ Overall, the data clearly show the same significant ‘deservingness gap’ between native citizens and immigrants that has been reported in several previous studies (e.g. Reeskens and van der Meer, 2019). Looking at the effects of the other vignette characteristics, we find that vignette persons are granted higher benefits if they lost their jobs due to reasons outside of their immediate control rather than for personal reasons (i.e. their job contradicted their religious values, the omitted baseline), if they are making active efforts at finding new work (compared with the baseline, no efforts), if they have paid taxes and contributions for longer, and if they have lower personal savings and are therefore more in need (Petersen, 2012; van Oorschot, 2006). We also find that older persons and women are granted more generous benefits, but the effect magnitudes here are not very large.

### The Effects of SDO, Ethnocentrism and Authoritarianism

**Figure 3.**
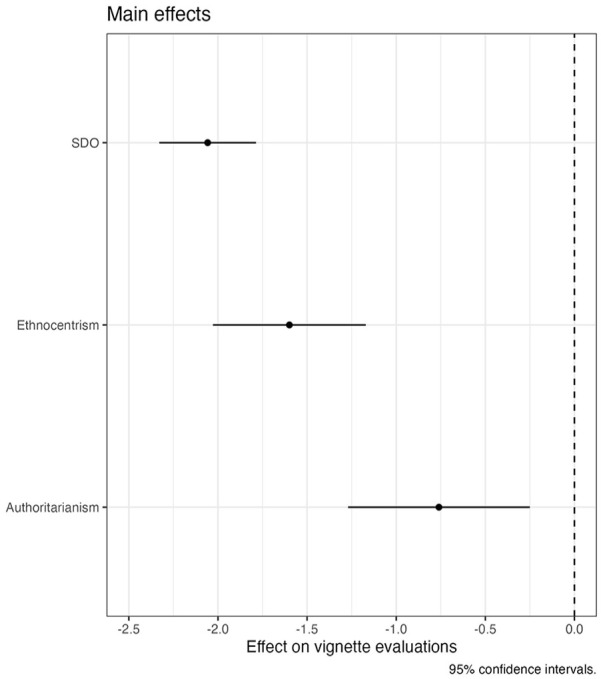
The Main Effects of SDO, Ethnocentrism and Authoritarianism on Deservingness Perceptions.

Next, we estimate a model in which we add the three core predictors that are available for all countries to the model to see if these have main effects on vignette evaluations. Figure 3 presents these main effects (see Table S-12 in the Online Appendix for the detailed results). All three variables have significant and negative effects, which means that persons who are more ethnocentric, more authoritarian or who score higher on SDO generally assign lower benefits to unemployed persons (immigrant or not).

Moving on to directly testing our first three hypotheses, we estimate the main interactive models, with which we test if bias against immigrants is conditioned by ethnocentrism (H1), authoritarianism (H2) or SDO (H3). Note that, as described earlier, we use different dichotomised versions of the immigrant-profile and interact them with authoritarianism, SDO and ethnocentrism, respectively.

**Figure 4.**
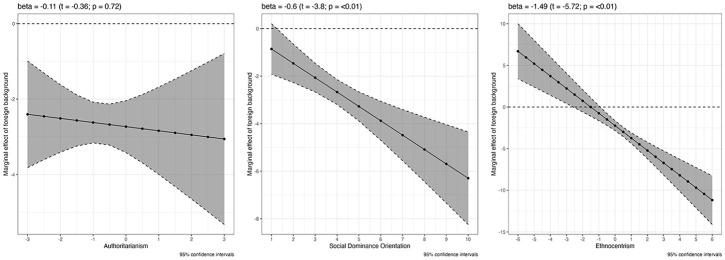
The Conditioning Effects of Psychological Predispositions on Vignette Ratings.

The three graphs in Figure 4 show the conditional effects of the psychological variables when we use the simple native citizen versus immigrant dummy (see also Table S-13 in the Online Appendix for the detailed results). As is immediately visible, and contrary to H1, authoritarianism does not have a significant conditioning effect. The effect of having an immigrant background is consistently negative and does become stronger as authoritarianism increases, but this increase is very slight and not statistically distinguishable from zero. SDO and ethnocentrism, by contrast, do have significant interactive effects in the expected negative direction, supporting H1 and H3. In the case of SDO, it is only at a very low score of 1 where no discrimination against foreign citizens takes place. Already persons that have a still relatively low SDO score of 3 exhibit significant bias against non-citizens and assign them replacement rates that are two percentage points lower than those assigned to citizens, on average and all other factors held constant. This negative bias doubles at SDO scores of around 6 and continues to increase significantly as SDO increases. Considering that most respondents in all countries fall into the range between 2 and 6 on the SDO scale (see Figure S-11 in the Online Appendix), this indicates that opposition to immigrants’ social rights is quite widespread in our samples. This result remains unchanged when we add ethnocentrism and authoritarianism as additional controls to the model (see Table S-14 in the Online Appendix).

The case of ethnocentrism is only seemingly different. Here, significant negative bias against non-citizens begins at ethnocentrism scores of around −0.5 and becomes stronger from there on. At the highest possible level of ethnocentrism, that is a score of 6, respondents assign non-citizens deservingness scores that are 10 percentage points lower than those of citizens, all other factors held constant. At negative ethnocentrism scores (indicating bias *in favour* of immigrants), respondents assign higher deservingness scores to non-citizens. However, this finding needs to be interpreted in light of the fact that only very few respondents actually have negative scores (see Figure S-10 in the Online Appendix). Across all countries, most respondents tend to fall in the neutral to positive range, which means that opposition to immigrants’ social rights is not confined to narrow groups of respondents. Controlling for SDO and authoritarianism does not change the conditioning effect of ethnocentrism (see Table S-14 in the Online Appendix).

**Figure 5.**
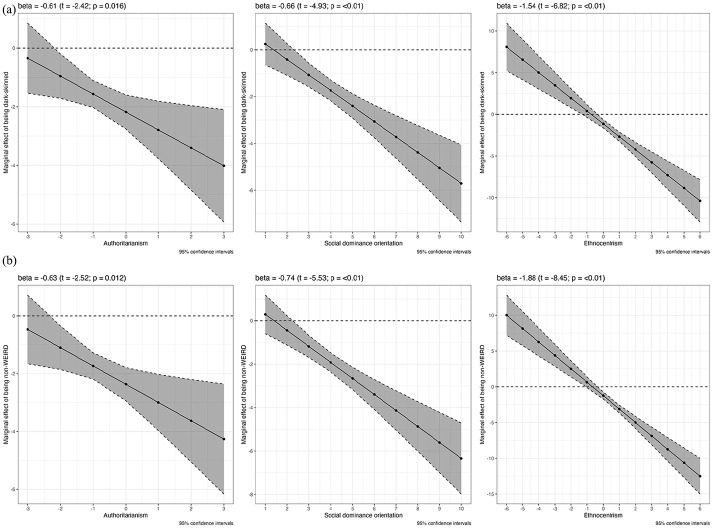
The Conditioning Effects of Psychological Predispositions on Vignette Ratings (Pooled Data; Alternative Dummy Coding). (a) Stereotypically Light- Versus Dark-Skinned. (b) WEIRD Versus Non-WEIRD.

We then replace the original immigrant/native citizen dummy with the one focused on skin colour (stereotypically light- or dark-skinned; see also above) and re-estimate the interactive models The main results (conditional marginal effects) are shown in Figure 5 (the detailed results are reported in Table S-17 in the Online Appendix). When looking at the top row, which shows the conditional effects of the psychological variables when using the light- versus dark-skinned dummy, we find again that SDO and ethnocentrism have significant conditioning effects: Persons stronger in these two variables discriminate more strongly against groups with (stereotypically) darker skin. However, an important difference is that we now also find a statistically significant effect of authoritarianism that goes in the expected direction: Persons who lean more strongly authoritarian also discriminate more strongly against groups with darker skin tones, all else being equal. This corresponds to the earlier findings by Crepaz (2020). The conditioning effect of authoritarianism is robust to adding SDO and ethnocentrism as controls (see Table S-17 in the Online Appendix). Overall, these results lend support to H2 and H3 (SDO and ethnocentrism) and now also to our first hypothesis (authoritarianism).

We get very similar results when we re-estimate the models using our third dummy (WEIRD vs non-WEIRD; shown in the bottom row of Figure 5).^25^ SDO, ethnocentrism and again authoritarianism have significant moderating effects. The latter results suggest that while SDO and ethnocentrism fuel opposition towards social rights for immigrants per se, the effect of authoritarianism is more limited to immigrant groups that are visibly or culturally different. This would be consistent with the idea that authoritarianism is primarily an aversion to diversity and difference, while SDO and ethnocentrism reflect, respectively, a brute desire to dominate or a sense of superiority over others, however close or distant these others may be.

### The Effect of Implicit Bias

Having established conditioning effects of three predictors, we now consider the effects of implicit bias (H4). The specification is the same as the one used above: We first interact the IAT *D*-scores with the dummy for foreign citizens while including all other vignette and respondent-level controls into the model. The only difference from the previous estimations is that we run the model separately on each sample, the reason for this being that we want to control for race and ethnicity in the US but lack equivalent data for Germany.^26^

**Figure 6.**
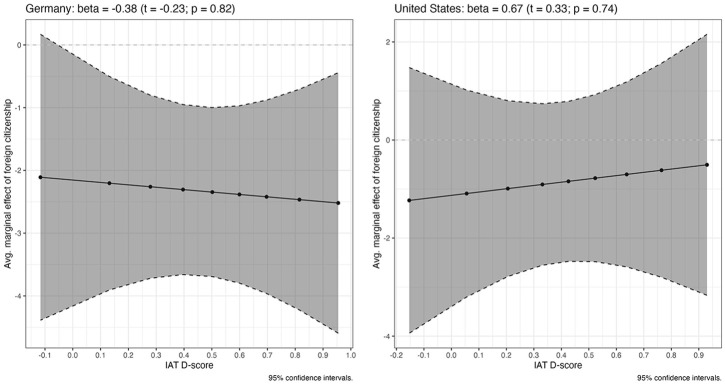
Implicit Bias Does Not Condition Attitudes Towards Immigrants’ Social Rights (Immigrant Vs Native Citizen Dummy).

The main results (using the immigrant vs native citizen dummy) are presented in Figure 6 (see Tables S-15 and S-16 in the Online Appendix for the detailed results) and point in both cases to the same conclusion: Implicit bias does not have a conditioning effect on attitudes towards immigrants’ social rights. We also do not find a main effect of the IAT *D*-scores on vignette evaluations.

Since our IAT measured implicit bias with respect to skin tone, one may theoretically expect stronger conditioning effects of implicit bias to emerge when we use the other two dummies, in particular the light- versus dark-skinned dummy (as also mentioned above). However, this is not borne out by the data. Implicit bias remains insignificant as a conditioning factor when we use the light- vs dark-skinned or WEIRD vs non-WEIRD dummies (see Figures S-15 and S-16 and the associated tables in the Online Appendix). This being said, we do note that the conditioning effect of implicit bias becomes visibly stronger and closer to statistical significance in the German data when using the WEIRD versus non-WEIRD dummy (see Figure S-16 in the Online Appendix). The pattern is very similar to those found for the other variables: Persons who do not have (or have positive) implicit bias towards darker-skinned persons do not discriminate against non-WEIRD immigrants – that is the effect of being non-WEIRD is not different from 0. At higher levels of implicit bias against darker-skinned persons, however, the effect of being non-WEIRD becomes different from 0 and then more strongly negative as implicit bias increases. The interaction term itself – capturing the *increase* in the effect magnitude as implicit bias becomes stronger – is not significant, however (see also Table S-21 in the Online Appendix). Therefore, we refrain from interpreting this finding as evidence for a conditioning effect of implicit bias. However, this null finding with respect to implicit bias does have an interesting implication: Opposition to the inclusion of immigrants into social protection systems does not appear to be driven by spontaneous and partly subconscious emotional responses. In other words, when people think of non-citizens as less deserving of support, they do so explicitly.

## Conclusion

Why do people oppose extending social rights to immigrants? Adding to previous research on this question, our results clarify the role of psychological predispositions for this attitude. The significant effects of ethnocentrism and SDO indicate that opposition to immigrants’ social rights is driven by a combination of negative stereotypes about immigrants and a desire for a hierarchically organised society in which a stronger in-group dominates others. In contrast, we find that a deep-seated unease about social and cultural diversity (as implied by authoritarianism) drives opposition to social rights for immigrants only when it comes to immigrants that are relatively distinct from the native mainstream population. Finally, we do not find that implicit bias plays a role.

Although only a peripheral part of our analysis, our results also add to our understanding of the socio-economic bases of opposition for immigrants’ social rights. Our data suggest that at least a part of those who want to exclude immigrants from the welfare state do not do so from a position of weakness and a desire for protection, but at least partially from a position of (economic) strength and a desire for dominance.

More generally, these results contribute also to the broader literature on right-wing populist politics, where opposition to immigrants’ social rights is a central ideological element (e.g. Andersen and Bjørklund, 1990; Careja et al., 2016). Resembling the literature on attitudes towards immigrants’ social rights, there is a tendency in the right-wing populism literature to attribute support for right-wing populist parties and movements as a response to relative economic decline and insufficient cultural resources to deal with societal change and increasing diversity (e.g. Inglehart and Norris, 2017; Kurer, 2020) – that is vulnerability and weakness. Our results do not directly contradict these accounts, but add the qualification that a desire for inequality, hierarchy and dominance may also be a part of the explanation (see also De Cleen and Ruiz Casado, 2023; Pettigrew, 2017).

Our study has limitations which could be addressed in future research. First, we acknowledge that our use of the generic skin-tone IAT instead of a more tailored one specifically on citizenship or immigration background might explain the absence of an effect of implicit bias, and it might therefore be worthwhile to conduct a new analysis using an IAT or similar instrument that captures implicit bias against immigrants more directly.

On a related note, another potential explanation for the absence of an effect of implicit bias may be that respondents suppressed these biases when subsequently evaluating the vignettes.^27^ It is known that even if people hold implicit attitudes, they may suppress them when responding to, for example, survey questions out of a desire to make a favourable impression (Nosek, 2005). However, we are not fully convinced that this is a likely explanation given that our vignette experiment should suppress social desirability bias (see above). Instead, we see it as more likely that what Nosek (2005: 566–567) called insufficient ‘evaluative strength’ is the culprit. Evaluative strength is the degree to which people see a given evaluation (in our case the vignette deservingness rating) as familiar, important and straightforward. The more this is the case, the more do people have automatic and encoded responses to it and thus stronger links of these explicit attitudes with underlying automatic implicit attitudes (Nosek, 2005: 567). It may be that respondents simply think too infrequently about welfare deservingness or social policy in general to have established automatic responses, which could explain the weak effect of implicit bias we find here and the stronger effects of explicit variables. In any case, we suspect that future research could establish a stronger link between implicit bias and explicit attitudes towards immigrants’ social rights by using a different experimental design that uses more closely corresponding implicit and explicit measures and which circumvents issues of lacking familiarity.

A second limitation is that our vignette experiment covers obviously only one particular type of social protection programme, unemployment benefits, and it is not self-evident that our findings will necessarily travel to other types of programmes. It would therefore be worthwhile to expand the analysis to other types of social benefit and also service programmes, and we do so to an extent in other contributions (Bonoli et al., 2023a, 2023b).

Finally, while we have provided new evidence on the roles of important psychological factors, we acknowledge that there are related variables and mechanisms that we have not considered but which could be investigated in future work. One theory we did not consider here is social identity theory (SIT) (Tajfel et al., 1971), a prominent explanation for various forms of in-group bias that has also been used as an explanation for antipathy towards immigrants (Eger, 2010). However, SIT is not an individual-level variable (our focus here) but more a general theory to explain bias against perceived outsiders in general, which is why we have not considered it here. This being said, SIT does have individual-level implications, specifically that greater bias against out-group members enhances people’s self-esteem (e.g. Aberson et al., 2000), and this implication could be tested in future research.

In focusing on authoritarianism, SDO, ethnocentrism and implicit bias, we have also stayed at the level of psychological variables that directly capture people’s perspectives on society and intergroup relations. However, these variables have been shown to be themselves influenced by the even more general Big Five personality traits, producing a causal chain that goes from general personality traits over variables such as authoritarianism and SDO to attitudes towards perceived out-groups (e.g. Ekehammar et al., 2004; Perry and Sibley, 2012). An avenue for future research would therefore be to go one step further back in this causal chain and include general personality traits into an analysis. Existing studies that have successfully linked the Big Five traits to attitudes towards immigrants in general (Gallego and Pardos-Prado, 2014) indicate that this would be a fruitful endeavour.

## Supplemental Material

sj-docx-1-psx-10.1177_00323217241228456 – Supplemental material for What Drives Opposition to Social Rights for Immigrants? Clarifying the Role of Psychological Predispositions Supplemental material, sj-docx-1-psx-10.1177_00323217241228456 for What Drives Opposition to Social Rights for Immigrants? Clarifying the Role of Psychological Predispositions by Carlo M Knotz, Alyssa M Taylor, Mia K Gandenberger and Juliana Chueri in Political Studies

## Notes

1. Much of this previous research used the term ‘welfare chauvinism’ to refer to opposition to immigrants’ social rights. However, as Careja and Harris (2022) recently pointed out, ‘welfare chauvinism’ is also used to refer to a combination of general support for the welfare state and an opposition to the inclusion of immigrants into welfare states. The existence of these two different definitions unfortunately renders the term ‘welfare chauvinism’ less useful, and we therefore use the formulation ‘opposition to social rights for immigrants’ (similar to Hooijer, 2021; and in line with the original definition by Andersen and Bjørklund, 1990: 212).

2. Studies have also found conditioning effects of a third group of macro-level variables, in particular the overall design of welfare state institutions (e.g. van der Waal et al., 2013). However, while our empirical design accounts for these macro-level variables (see below), our focus here is squarely on the micro-level and the considerations behind people’s attitudes towards immigrants’ social rights. We explore the effects of welfare state institutions in our other work (Bonoli et al., 2023a, 2023b).

3. Social identity theory (SIT) (Tajfel et al., 1971) or the Big Five personality traits (MacCrae and Costa, 1999) could in principle also be named here since some previous studies have used it as their theoretical foundation (e.g. Eger, 2010) or have linked them to attitudes towards immigrants (Gallego and Pardos-Prado, 2014). We do not directly consider these explanations here, a choice which we discuss in the concluding section.

4. There is also a great amount of related evidence linking authoritarianism to anti-immigrant attitudes and political actors espousing them (e.g. MacWilliams, 2016; Pettigrew, 2017; Thomsen et al., 2008) as well as to greater opposition to inclusive, compassionate and generous social protection policies (e.g. Achterberg et al., 2014; Jedinger and Kaminski, 2023).

5. The theory of implicit cognition and attitudes has initially sparked some controversy, with some questioning the validity of measurements of implicit attitudes (e.g. Brendl et al., 2001) or the underlying theory more fundamentally (e.g. Arkes and Tetlock, 2004). However, a vast number of studies have since provided systematic evidence that implicit attitudes and biases exist and can be measured with available instruments (e.g. Nosek et al., 2007; see also below), that implicit attitudes are distinct from explicitly held views, and that they have significant effects on other attitudes and behaviour (e.g. Greenwald et al., 2009; Nosek et al., 2007; Pérez, 2013).

6. Ethics approval for the survey and the experiments was granted by the Research Ethics Commission of the University of Lausanne (Approval No. C_FDCA_022020_0001).

7. Germany: *N* = 3073; United States: *N* = 2932; United Kingdom: *N* = 1445; Switzerland: *N* = 1462; Sweden: *N* = 1457; Denmark: *N* = 1471.

8. See Figures S-1 through S-6 in the Online Appendix for the demographic composition of our samples.

9. The underlying formal experimental design used to ‘populate’ the vignettes was a *D*-efficient design (Kuhfeld, 2010). Using a *D*-efficient design rather than pure random sampling to generate the vignettes is generally recommended, but especially so when designs are relatively complex due to a larger number of attributes and excluded combinations (Auspurg and Hinz, 2015: 35). For *D*-efficiency scores 90 is the recommended threshold that a suitable design should achieve (Auspurg and Hinz, 2015). Our design achieves a score of 94.79 and does not exclude combinations of vignette attributes.

10. We dropped respondents who rated any single vignette in less than 5 seconds, which we see as implausibly quick.

11. We fielded the survey in 2021, thus respondents’ perceptions of Ukrainians could not yet have been influenced by the Russia-Ukraine war that started in early 2022. Ukraine was chosen as an origin country because it was, at the time, a relatively ‘generic’ non-European Union (EU) post-Soviet country and not associated with very pronounced country-specific stereotypes (unlike, e.g. Russia).

12. We calculate the average of the scores given to the immigrant groups on each dimension. This measures how intelligent, trustworthy and hard-working a given respondent perceives immigrants in general. Then we subtract that average score from the score given to the native-born. This measures to what extent a given respondent sees immigrants as better or worse than the native-born on each dimension. Finally, the differences are averaged over all three dimensions to obtain a general indicator of ethnocentrism.

13. In the calculation of the indicator, the egalitarian items (‘We must consider all groups’, ‘Group equality should be our ideal’) were reversed so that higher scores indicate greater social dominance orientation.

14. See Figures S-11 and S-12 in the Online Appendix.

15. Following some early criticism of the Implicit Association Test (IAT) as a valid measurement of implicit attitudes (e.g. Brendl et al., 2001), it has since been extensively validated and is nowadays widely used (Nosek et al., 2005, 2007; Nosek and Smyth, 2007; see also Glaser and Finn, 2013; Pérez, 2013 for reviews).

16. In the US, 1398 out of 2932 respondents completed the IAT. In Germany, this amounted to 1518 out of 3073 respondents.

17. We use IAT stimuli from *Project Implicit* (Nosek et al., 2007), retrieved from https://www.projectimplicit.net/nosek/stimuli/ and the *Project Implicit* demonstration tests, and Brendl et al. (2001). To arrive at equal numbers of targets and categories without accidentally contaminating the stimuli with our own potential biases, we drew random samples out of the entire pool of available stimuli. We list our target categories in the Online Appendix (Table S-1).

18. We used the ‘improved’ scoring procedure developed by Greenwald et al. (2003).

19. There is also evidence that immigrants are implicitly associated with darker skin tones, and that this implicit association is linked to explicit attitudes about immigrants (Zhirkov, 2023). The effects of implicit skin-tone bias may therefore also extend to all our immigrant groups.

20. We do not control for the respondent’s immigrant background in all models that include ethnocentrism, since this variable is only computed for respondents that were born in their country of residence (see also above).

21. When we rerun these analyses country-by-country (results are available upon request), we find some variation in these associations: Notably, SDO is positively linked to financial resources in all countries, but insignificantly so in Denmark and the United Kingdom. Likewise, the positive link between ethnocentrism and unemployment is driven by the United Kingdom. The negative associations between education and ethnocentrism, SDO and authoritarianism, finally, is particularly strong in Sweden but of more varying strength and often insignificant in the other countries.

22. The vignette-citizenship variable has five categories, but each respondent rated only three vignettes. Therefore, not all respondents actually saw all different nationalities, which makes it impossible to estimate the variation in the effects of different nationalities across all respondents (the random effects term can be thought of as a type of interaction between the respondent ID and the lower-level variable, the estimation of which requires observations of all lower-level variables for all respondents; see also Bates et al., 2015: 6). Entering a random effects term becomes possible when using a simplified immigrant-dummy.

23. The full results, including the coefficients for the respondent-variables and country dummies, are shown in Table S-11 in the Online Appendix.

24. Neighbouring versus Afghan: Difference = 1.6 (*t* = 4.43); Ukrainian versus Afghan: Difference = 1.37 (*t* = 3.88); Nigerian versus Afghan: Difference = −1.01 (*t* = −2.84).

25. See Table S-18 in the Online Appendix for the detailed results.

26. We did not include a question about the respondent’s race or ethnicity into the German questionnaire since race or skin colour are, for historical reasons, sensitive issues in Germany and we wanted to avoid irritating respondents.

27. We are grateful to one of the reviewers for pointing this out.
