# Supplementary material for: What Drives Opposition to Social Rights for Immigrants? Clarifying the Role of Psychological Predispositions
Source: Polit Stud (Oxf). 2024 Feb 12;73(1):101–25. doi: 10.1177/00323217241228456 (PMC11802323; doi:10.1177/00323217241228456)
Supplement: sj-docx-1-psx-10.1177_00323217241228456 – Supplemental material for What Drives Opposition to Social Rights for Immigrants? Clarifying the Role of Psychological Predispositions [file sj-docx-1-psx-10.1177_00323217241228456.docx]

What drives opposition to social rights for immigrants?

Clarifying the role of psychological predispositions

*Online Appendix*

Carlo Knotz^∗^, Alyssa Taylor, Mia Gandenberger, Juliana Chueri

December 20, 2023

# Contents

1. [Sample demographics](#_bookmark0) 4
2. [Psychological variables: Measurement, distributions & correlates](#_bookmark7) 10
3. [Vignette evaluations](#_bookmark25) 22
4. [Detailed estimation results (main analyses)](#_bookmark27) 23
   1. [Baseline models](#_bookmark28) 23
   2. [Interactive models (immigrant vs. non-immigrant)](#_bookmark31) 26
5. [Alternative codings of immigrant attribute](#_bookmark36) 31
   1. [Light- vs. dark-skinned](#_bookmark37) 31
   2. [WEIRD vs. Non-WEIRD](#_bookmark39) 33

∗[carlo.knotz@uis.no](mailto:carlo.knotz@uis.no)

# List of Figures

- 1. [Respondent demographics, Switzerland](#_bookmark1) 4
  2. [Respondent demographics, Germany](#_bookmark2) 5
  3. [Respondent demographics, Denmark](#_bookmark3) 6
  4. [Respondent demographics, Sweden](#_bookmark4) 7
  5. [Respondent demographics, United Kingdom](#_bookmark5) 8
  6. [Respondent demographics, United States](#_bookmark6) 9
  7. [IAT *D*-scores](#_bookmark9) 11
  8. [Implicit bias against dark-skinned persons increases with age](#_bookmark10) 12
  9. [Authoritarianism](#_bookmark11) 13
  10. [Ethnocentrism](#_bookmark12) 14
  11. [Social dominance orientation](#_bookmark13) 15
  12. [Males are higher in SDO than females](#_bookmark14) 16
  13. [Socio-economic correlates of psychological variables (pooled)](#_bookmark21) 19
  14. [The distributions of vignette evaluations across countries](#_bookmark26) 22
  15. [Implicit bias does not have a conditioning effect: Alternative coding](#_bookmark41)

[of immigrant attribute (light- vs. dark-skinned)](#_bookmark41) 35

- 1. [Implicit bias does not have a conditioning effect: Alternative coding](#_bookmark44)

[of immigrant attribute (WEIRD vs. Non-WEIRD)](#_bookmark44) 38

**List of Tables**

- 1. [IAT stimuli](#_bookmark8) 10
  2. [Correlations between core independent variables (Switzerland)](#_bookmark15) 17
  3. [Correlations between core independent variables (Germany)](#_bookmark16) 17
  4. [Correlations between core independent variables (Denmark)](#_bookmark17) 17
  5. [Correlations between core independent variables (Sweden)](#_bookmark18) 17
  6. [Correlations between core independent variables (United Kingdom)](#_bookmark19) 17
  7. [Correlations between core independent variables (United States)](#_bookmark20) 18
  8. [The demographic correlates of ethnocentrism, SDO, and authori-](#_bookmark22) [tarianism](#_bookmark22) 20
  9. [The demographic correlates of implicit bias (US)](#_bookmark23) 21
  10. [The demographic correlates of implicit bias (DE)](#_bookmark24) 21
  11. [Baseline model (pooled)](#_bookmark29) 24
  12. [Main effects of personality variables (pooled)](#_bookmark30) 25
  13. [Main interactive models (pooled)](#_bookmark32) 27
  14. [Interactive models – robustness checks (pooled)](#_bookmark33) 28
  15. [Models of effects of IAT *D*-scores (US)](#_bookmark34) 29
  16. [Models of effects of IAT *D*-scores (DE)](#_bookmark35) 30
  17. [Interactive models – Alternative immigrant coding (light-vs. dark-](#_bookmark38) [skinned; pooled)](#_bookmark38) 32
  18. [Interactive models – Alternative immigrant coding (WEIRD vs.](#_bookmark40)

[Non-WEIRD; pooled)](#_bookmark40) 34

- 1. [Models of effects of IAT *D*-scores (DE) – alternative immigrant](#_bookmark42) [attribute coding (light- vs. dark-skinned)](#_bookmark42) 36
  2. [Models of effects of IAT *D*-scores (US) – alternative immigrant at-](#_bookmark43) [tribute coding (light- vs. dark-skinned)](#_bookmark43) 37
  3. [Models of effects of IAT *D*-scores (DE) – alternative immigrant](#_bookmark45) [attribute coding (WEIRD vs. Non-WEIRD)](#_bookmark45) 39
  4. [Models of effects of IAT *D*-scores (US) – alternative immigrant at-](#_bookmark46) [tribute coding (WEIRD vs. Non-WEIRD)](#_bookmark46) 40

# Sample demographics

50

40

100

30

Percent

Observations

20 50

10

0

Female

1. Gender

Male

0

25 50 75

Respondent's age

1. Age

100

20 bins

Tertiary (BA, MA, Professional, PhD) 60

40

Percent

Upper sec./post−sec. non−tertiary

20

None/primary/lower sec.

0

0 10 20 30 40

Percent

1. Education

Urban Rural

1. Geographic area

Figure S-1: Respondent demographics, Switzerland

*Notes:* Urban: A big city, suburbs/outskirts, town or small city; rural: Country village, farm or home in countryside.

50

300

40

200

Observations

30

Percent

20

100

10

0

Female

1. Gender

Male

0

30 50 70

Respondent's age

1. Age

90

20 bins

80

Tertiary (BA, MA, Professional, PhD)

60

Upper sec./post−sec. non−tertiary 40

Percent

20

None/primary/lower sec.

0

0 10 20 30 40 50

Percent

1. Education

Urban Rural

1. Geographic area

Figure S-2: Respondent demographics, Germany

*Notes:* Urban: A big city, suburbs/outskirts, town or small city; rural: Country village, farm or home in countryside.

150

50

40

100

30

Percent

Observations

20

50

10

0

Female

1. Gender

Male

0

25 50 75

Respondent's age

1. Age

100

20 bins

80

Tertiary (BA, MA, Professional, PhD)

60

Upper sec./post−sec. non−tertiary

Percent

40

20

None/primary/lower sec.

0

0 10 20 30 40

Percent

1. Education

Urban Rural

1. Geographic area

Figure S-3: Respondent demographics, Denmark

*Notes:* Urban: A big city, suburbs/outskirts, town or small city; rural: Country village, farm or home in countryside.

50 150

40

100

30

Percent

Observations

20

50

10

0

Female

1. Gender

Male

0

25 50 75

Respondent's age

1. Age

20 bins

80

Tertiary (BA, MA, Professional, PhD)

60

Upper sec./post−sec. non−tertiary

Percent

40

20

None/primary/lower sec.

0

0 10 20 30 40

Percent

1. Education

Urban Rural

1. Geographic area

Figure S-4: Respondent demographics, Sweden

*Notes:* Urban: A big city, suburbs/outskirts, town or small city; rural: Country village, farm or home in countryside.

120

50

40 90

30

Observations

60

Percent

20

30

10

0

Female

1. Gender

Male

0

25 50 75

Respondent's age

1. Age

100

20 bins

80

Tertiary (BA, MA, Professional, PhD)

60

Upper sec./post−sec. non−tertiary

Percent

40

20

None/primary/lower sec.

0

0 10 20 30 40

Percent

1. Education

Urban Rural

1. Geographic area

Figure S-5: Respondent demographics, United Kingdom

*Notes:* Urban: A big city, suburbs/outskirts, town or small city; rural: Country village, farm or home in countryside.

300

50

Tertiary (BA, MA, Professional, PhD)

40

200

30

Percent

Observations

Upper sec./post−sec. non−tertiary

20 100

10

None/primary/lower sec.

0

Female

Male

0

25 50 75

Respondent's age

100

20 bins

0 10

20 30

Percent

40 50

1. Gender
2. Age
3. Education

80

80

60

60

40

Percent

Percent

40

20

20

0 0

Urban Rural White Black/AfrAm Asian/PI Hispanic Other

1. Geographic area (e) Race/ethnicity

Figure S-6: Respondent demographics, United States

*Notes:* Urban: A big city, suburbs/outskirts, town or small city; rural: Country village, farm or home in countryside. Race/ethnicity: “Other” includes “American Indian or Alaska Native”, “Middle Eastern or North African”, and respondents who placed themselves in the “Other” cat- egory. “Asian/PI” includes “Chinese”, “Vietnamese”, “Japanese”, “Korean”, “Filipino”, “Asian India”, “Other Asian”, “Native Hawaiian”, “Samoan”, “Chamorro”, “Other Pacific Islander”. “Hispanic” includes “Mexican, Mexican American, Chicano”, “Puerto Rican”, “Cuban”, and “Another Hispanic, Latino, Spanish origin”.

# Psychological variables: Measurement, distributions & correlates

Objects Stimuli

*Categories*

United States “Good” Glorious, Lovely, Cheer, Beautiful,

Delight, Cherish, Laughing, Pleasing “Bad” Hurtful, Scorn, Dirty, Disgust, Ugly,

Failure, Rotten, Poison

Germany “Good” Glu¨cklich, Frieden, Vergnu¨gen, Prachtvoll,

Liebe, Lachen, Freude, Wundervoll “Bad” Qual, Verletzt, Misserfolg, B¨ose, U¨ bel,

Schrecklich, Grausam, Scheußlich

*Targets* Light skin tone Images (“light”)

Dark skin tone Images (“dark”)

Table S-1: IAT stimuli

*Notes:* The target images are available from

<https://www.projectimplicit.net/nosek/stimuli/>(last access on April 27, 2022).

250

250

15000

200 200

10000

150 150

Count

Count

Count

100 100

5000

50 50

0 0 0

−1 0 1 2

IAT D−scores

Number of bins set to 30.

- 1. Germany

−2 −1 0 1 2

IAT D−scores

Number of bins set to 30.

- 1. United States

−2 −1 0 1 2

IAT D−scores

Number of bins set to 30.

- 1. Comparison data

Figure S-7: IAT *D*-scores

*Note:* The Project Implicit data shown in panel (c) are *D*-scores from skin-tone IATs adminis- tered via the project’s demonstration website ([https://implicit.harvard.edu](https://implicit.harvard.edu/)) in 2015.

2


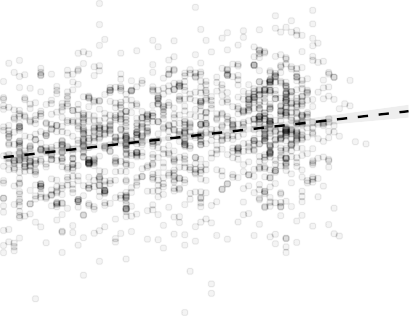


|  |  |  |  |  |  |  |  |
| --- | --- | --- | --- | --- | --- | --- | --- |
|  |  |  |  |  |  |  |  |
|  |  |  |  |  |  |  |  |
|  |  |  |  |  |  |  |  |
|  |  |  |  |  |  |  |  |
|  |  |  |  |  |  |  |  |
|  |  |  |  |  |  |  |  |
|  |  |  |  |  |  |  |  |

2

1 1

IAT D−Score

IAT D−Score

0

0

−1

−1

25 50 75 100

Respondent's age

Pearson corr. = 0.23 (p < 0.01)

- - 1. United States


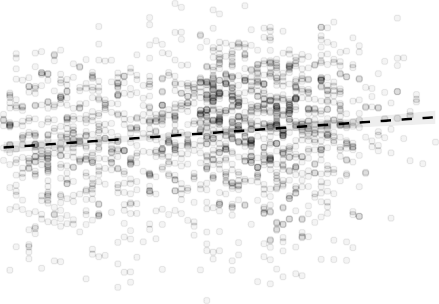


30 50 70

Respondent's age

Pearson corr. = 0.13 (p < 0.01)

- - 1. Germany

Figure S-8: Implicit bias against dark-skinned persons increases with age

*Notes:* This replicates the finding by [Nosek et al.](#_bookmark49) ([2007](#_bookmark49), 32).

50 50

40 40

30 30

Percent

Percent

20 20

10 10

0 0

−3 −2 −1 0 1 2 3

Authoritarianism score

- - - 1. Germany

50

40

30

Percent

20

10

0

−3 −2 −1 0 1 2 3

Authoritarianism score

- - 1. Switzerland

−3 −2 −1 0 1 2 3

Authoritarianism score

(b) United States

50

|  |  |  |  |  |  |  |  |  |  |  |  |  |  |  |  |
| --- | --- | --- | --- | --- | --- | --- | --- | --- | --- | --- | --- | --- | --- | --- | --- |
|  |  |  |  |  |  |  |  |  |  |  |  |  |  |  |  |
|  |  |  |  |  |  |  |  |  |  |  |  |  |  |  |  |
|  |  |  |  |  |  | |  |  |  |  |  |  |  |  |  |
|  |  |  |  |  |  |  |  |  |  |  |  |  |  |  |  |
|  |  |  |  |  |  |  |  | |  |  |  |  |  |  |  |
|  |  |  |  |  |  |  |  |  |  |  |  |  |  |  |  |
|  |  |  |  |  |  |  |  |  |  |  |  |  |  |  |  |
|  |  |  |  |  |  |  |  |  |  |  |  |  |  |  |  |
|  |  |  |  | |  |  |  |  |  |  |  |  |  |  |  |
|  |  |  |  |  |  |  |  |  |  |  |  |  |  |  |  |
|  |  |  |  |  |  |  |  |  |  | |  |  |  |  |  |
|  |  |  |  |  |  |  |  |  |  |  |  |  |  |  |  |

40

Percent

30

20

10

0

−3 −2 −1 0 1 2 3

Authoritarianism score

- - 1. United Kingdom

50 50

40 40

30 30

Percent

Percent

20 20

10 10

0 0

−3 −2 −1 0 1 2 3

Authoritarianism score

- - 1. Sweden

−3 −2 −1 0 1 2 3

Authoritarianism score

- - 1. Denmark

Figure S-9: Authoritarianism

*Notes:* See also [Crepaz](#_bookmark47) ([2020](#_bookmark47), 1258) or [Tillman](#_bookmark51) ([2013](#_bookmark51), 575) for corresponding findings.

1200

900

900

600

Count

Count

600

300

300

0 0

−2.5 0.0 2.5 5.0

Ethnocentrism

Number of bins set to 30.

- - - 1. Germany

−6 −3 0 3 6

Ethnocentrism

Number of bins set to 30.

- - - 1. United States

300

400

200

Count

Count

200

100

0 0

−5.0 −2.5 0.0 2.5 5.0

Ethnocentrism

Number of bins set to 30.

- - - 1. Switzerland

−2.5 0.0 2.5 5.0

Ethnocentrism

Number of bins set to 30.

- - - 1. United Kingdom

400

400

300

300

Count

Count

200

200

100 100

0 0

−3 0 3 6

Ethnocentrism

Number of bins set to 30.

- - - 1. Sweden

−2.5 0.0 2.5 5.0

Ethnocentrism

Number of bins set to 30.

- - - 1. Denmark

Figure S-10: Ethnocentrism

*Notes:* The original item scales that were presented in the questionnaires ranged from 1 (“trustworthy”/“intelligent”/“hard-working”) to 7 (“untrustworthy”/“intelligent”/“lazy”). The scores were reversed in the analysis so that higher scores now indicate more positive attributes. Ethnocentrism scores were only computed for respondents that reported that they were born in their respective countries of residence. See also [Kam and Kinder](#_bookmark48) ([2012](#_bookmark48), 328).

400

300

200

Count

100

0


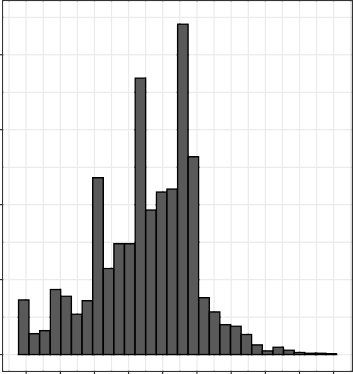
1 2 3 4 5 6 7 8 9 10

Social dominance orientation

Number of bins set to 30.

1. Germany

400

300

200

Count

100

0


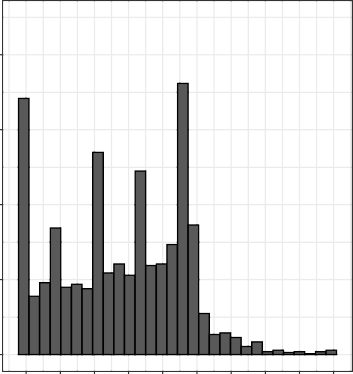
1 2 3 4 5 6 7 8 9 10

Social dominance orientation

Number of bins set to 30.

1. United States

250

200

150

Count

100

50

0

250

200

150

Count

100

50

0


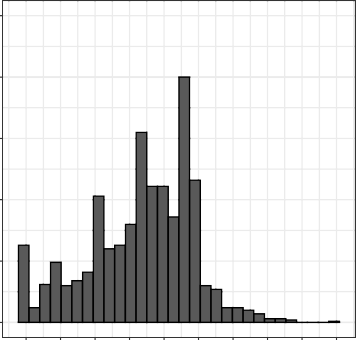


1 2 3 4 5 6 7 8 9 10

Social dominance orientation

Number of bins set to 30.

1. Switzerland


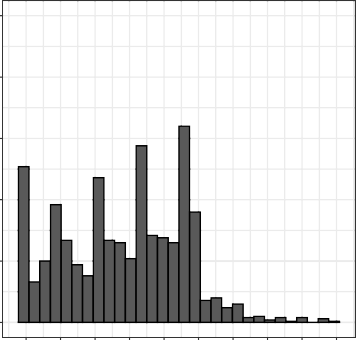


1 2 3 4 5 6 7 8 9 10

Social dominance orientation

Number of bins set to 30.

(e) Sweden

250

200

150

Count

100

50

0

250

200

150

Count

100

50

0


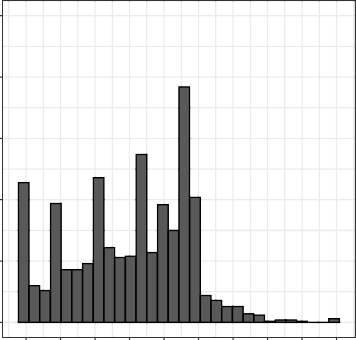


1 2 3 4 5 6 7 8 9 10

Social dominance orientation

Number of bins set to 30.

1. United Kingdom


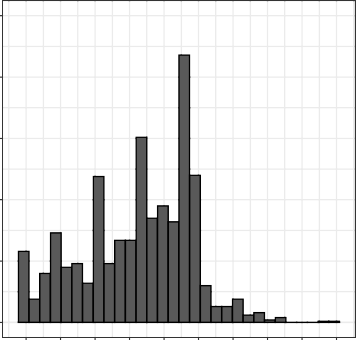


1 2 3 4 5 6 7 8 9 10

Social dominance orientation

Number of bins set to 30.

- 1. Denmark

Figure S-11: Social dominance orientation

*Notes:* The response scale ranged from 1 (“Oppose extremely”) to 10 (“Favor extremely”). The SDO score is the average over all item scores, where the two pro-egalitarian items (“In setting priorities, we must consider all groups” & “Group equality should be our ideal”) were reverse- coded so that higher scores indicate a greater social dominance orientation.


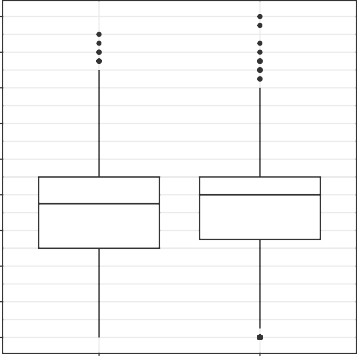
10 10

9 9

8 8

7 7

Social dominance orientation

Social dominance orientation

6 6

5 5

4 4

3 3

2 2

1 1

Female Male

Gender

Mean males = 4.6; Mean females = 4.4;

Female Male

Gender

Mean males = 4.1; Mean females = 3.5;

- - 1. Germany

(t = −4.03; p−value: <0.001

(t = −8.28; p−value: <0.001

- - 1. United States

10 10

9 9

8 8

7 7

Social dominance orientation

Social dominance orientation

6 6

5 5

4 4

3 3

2 2

1 1

Female Male

Gender

Mean males = 4.5; Mean females = 4.2;

Female Male

Gender

Mean males = 4.2; Mean females = 3.8;

- - 1. Switzerland

(t = −2.8; p−value: 0.00512

(t = −5.31; p−value: <0.001

- - 1. United Kingdom

10 10

9 9

8 8

7 7

Social dominance orientation

Social dominance orientation

6 6

5 5

4 4

3 3

2 2

1 1

Female Male

Gender

Mean males = 4.1; Mean females = 3.6;

Female Male

Gender

Mean males = 4.5; Mean females = 4;

- - 1. Sweden

(t = −5.48; p−value: <0.001

- - 1. Denmark

(t = −6.22; p−value: <0.001

Figure S-12: Males are higher in SDO than females

*Notes:* This replicates the finding by [Pratto et al.](#_bookmark50) ([1994](#_bookmark50), 747). See also Figure [S-11](#_bookmark13) above for details on the SDO measure.

Authoritarianism Ethnocentrism

| Ethnocentrism | 0.02 |  |
| --- | --- | --- |
| SDO | 0.14* | 0.07* |
| * *p <* 0*.*05 |  |  |

Table S-2: Correlations between core independent variables (Switzerland)

Authoritarianism Ethnocentrism SDO

| Ethnocentrism | 0.08* |  |  |
| --- | --- | --- | --- |
| SDO | 0.14* | 0.13* |  |
| D-Score | 0.00 | 0.08* | 0.04 |

* *p <* 0*.*05

Table S-3: Correlations between core independent variables (Germany)

Authoritarianism Ethnocentrism

| Ethnocentrism | 0.06* |  |
| --- | --- | --- |
| SDO | 0.32* | 0.18* |
| * *p <* 0*.*05 |  |  |

Table S-4: Correlations between core independent variables (Denmark)

Authoritarianism Ethnocentrism

| Ethnocentrism | 0.05 |  |
| --- | --- | --- |
| SDO | 0.28* | 0.17* |
| * *p <* 0*.*05 |  |  |

Table S-5: Correlations between core independent variables (Sweden)

Authoritarianism Ethnocentrism

| Ethnocentrism | 0.06 |  |
| --- | --- | --- |
| SDO | 0.27* | 0.18* |
| * *p <* 0*.*05 |  |  |

Table S-6: Correlations between core independent variables (United Kingdom)

Authoritarianism Ethnocentrism SDO

| Ethnocentrism | 0.12* |  |  |
| --- | --- | --- | --- |
| SDO | 0.28* | 0.15* |  |
| D-Score | -0.01 | 0.07* | 0.02 |

* *p <* 0*.*05

Table S-7: Correlations between core independent variables (United States)

Unemployed

*

*

~~*~~

*

*

*

* *

* * *

*

**

Savings

Male

Tert. educ.

Sec. educ.

Born in country

Age

−0.4 −0.2 0.0 0.2

Effect estimate

Authorit. Ethnocent. SDO

95% confidence intervals. Country−fixed effects omitted. * p < 0.05

- - - 1. Ethnocentrism, authoritarianism, and SDO

Unemployed

Unemployed

Savings

Other

Savings

Hispanic

Asian/Pac.Isl.

Male

Black/Afr.Amer. *

Tertiary (BA, MA, Professional, PhD)

Male

Tertiary (BA, MA, Professional, PhD)

Upper sec./post−sec. non−tertiary

Upper sec./post−sec. non−tertiary

Born in country

Born in country

Age *

Age *

−0.25 0.00

Effect estimate

* p<0.05; 95% confidence intervals

- - - 1. Implicit bias (US)

−0.2 −0.1 0.0

Effect estimate

* p<0.05; 95% confidence intervals

- - - 1. Implicit bias (DE)

Figure S-13: Socio-economic correlates of psychological variables (pooled)

*Notes:* See Tables [S-8](#_bookmark22), [S-9](#_bookmark23), and [S-10](#_bookmark24) for the detailed results.

|  | SDO | Ethnocentrism | Authoritarianism |
| --- | --- | --- | --- |
| Intercept  Country dummies | 3*.*75^∗^ (0*.*09) | 0*.*29^∗^ (0*.*06) | *−*0*.*85^∗^  (0*.*05) |

Germany 0*.*13^∗^ 0*.*07 0*.*16^∗^ (0*.*06) (0*.*04) (0*.*03)

*— −*

Denmark 0*.*32^∗^ 0*.*16^∗^ 0*.*06

*— −*

(0*.*07) (0*.*05) (0*.*04)

Sweden 0*.*75^∗^ 0*.*05 0*.*03

*— −*

(0*.*07) (0*.*05) (0*.*04)

United Kingdom 0*.*54^∗^ 0*.*48^∗^ 0*.*10^∗^ (0*.*07) (0*.*05) (0*.*04)

*— −*

United States 0*.*64^∗^ 0*.*53^∗^ 0*.*43^∗^ (0*.*06) (0*.*04) (0*.*03)

*— −*

Resp. demographics

| Age | 0*.*01^∗^ | 0*.*01^∗^ | 0*.*00^∗^ |
| --- | --- | --- | --- |
| Upper sec./post-sec. non.-tert. | (0*.*00)  *−*0*.*16^∗^ | (0*.*00)  *−*0*.*13^∗^ | (0*.*00)  *−*0*.*11^∗^ |

(0*.*05) (0*.*03) (0*.*03)

Tertiary (BA, MA, Professional) 0*.*29^∗^ 0*.*25^∗^ 0*.*24^∗^

*— − −*

(0*.*05) (0*.*03) (0*.*03)

Male 0*.*29^∗^ 0*.*06^∗^ 0*.*03

(0*.*03) (0*.*02) (0*.*02)

Born in country 0*.*07 0*.*13^∗^

*−*

(0*.*06) (0*.*03)

Unemployed *−*0*.*15^∗^ 0*.*11^∗^ *−*0*.*07

| (0*.*07) | | (0*.*05) | (0*.*04) |
| --- | --- | --- | --- |
| Savings 0*.*07^∗^ | | 0*.*01 | 0*.*01 |
| (0*.*01) | | (0*.*01) | (0*.*01) |
| R^2^ 0*.*07 | | 0*.*09 | 0*.*06 |
| Num. obs. | 9399 | 8511 | 9399 |
| F statistic | 61*.*19 | 73*.*06 | 51*.*08 |
| * *p <* 0*.*05; standard errors in parentheses. |  |  |  |

Table S-8: The demographic correlates of ethnocentrism, SDO, and authoritari- anism

IAT D-score

Intercept 0*.*21 (0*.*10)^∗^

Resp. age 0*.*00 (0*.*00)^∗^

Resp. not US-born 0*.*10 (0*.*05)

Upper sec./post-sec. non-tertiary 0*.*02 (0*.*10) Tertiary (BA, MA, Professional, PhD) 0*.*00 (0*.*10) Male 0*.*00 (0*.*03)

*−*

∗

*−*

Black/Afr.Amer. 0*.*36 (0*.*05)

*−*

Asian/Pac.Isl. 0*.*09 (0*.*06)

*−*

Hispanic 0*.*08 (0*.*08)

*−*

Other 0*.*13 (0*.*07)

*−*

Available savings 0*.*01 (0*.*01)

Unempl. 0*.*00 (0*.*04)

R^2^ 0*.*10

Num. obs. 1119

F statistic 10*.*72

* *p <* 0*.*05; standard errors in parentheses.

Table S-9: The demographic correlates of implicit bias (US)

IAT D-score

Intercept 0*.*29 (0*.*05)^∗^

Resp. age 0*.*00 (0*.*00)^∗^

Resp. not DE-born 0*.*05 (0*.*05)

*−*

Upper sec./post-sec. non-tertiary 0*.*02 (0*.*03) Tertiary (BA, MA, Professional, PhD) 0*.*03 (0*.*03) Male 0*.*04 (0*.*02)

*−*

*−*

Available savings 0*.*01 (0*.*01)

Unempl. *−*0*.*09 (0*.*06)

R^2^ 0*.*03

Num. obs. 1249

F statistic 4*.*77

* *p <* 0*.*05; standard errors in parentheses.

Table S-10: The demographic correlates of implicit bias (DE)

# Vignette evaluations


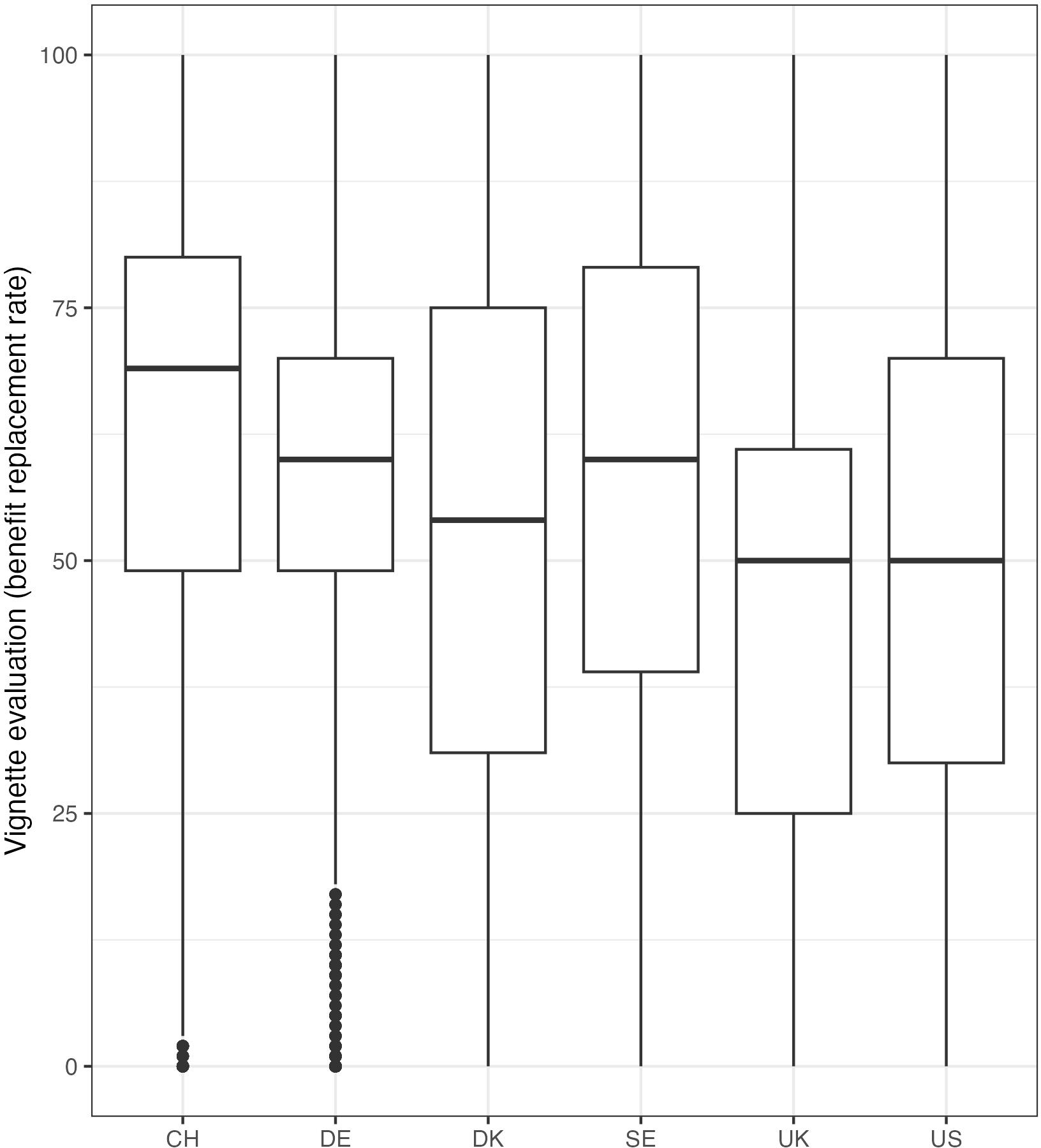


Figure S-14: The distributions of vignette evaluations across countries

# Detailed estimation results (main analyses)

## Baseline models

Starts overleaf.

Baseline model

Intercept 57*.*01 (1*.*22)^∗^

Vignette attributes

Female 0*.*94 (0*.*21)^∗^

Age: 40 years 0*.*54 (0*.*26)^∗^

Age: 55 years 1*.*29 (0*.*26)^∗^

Commute too long 5*.*74 (0*.*31)^∗^

Excessive overtime 5*.*29 (0*.*30)^∗^

Closure due to COVID 15*.*23 (0*.*30)^∗^

Tax/Contr: 2 years 1*.*05 (0*.*30)^∗^

Tax/Contr: 4 years 1*.*46 (0*.*30)^∗^

Tax/Contr: 8 years 2*.*88 (0*.*30)^∗^

Residency: 10 years 1*.*43 (0*.*30)^∗^

*−*

Residency: 5 years 2*.*24 (0*.*30)^∗^

*−*

Residency: 2 years 3*.*44 (0*.*30)^∗^

*−*

Neighboring 2*.*05 (0*.*35)^∗^

*−*

Ukrainian 2*.*29 (0*.*34)^∗^

*−*

Afghan 3*.*66 (0*.*35)^∗^

*−*

Nigerian 2*.*65 (0*.*34)^∗^

*−*

Lab technician 0*.*32 (0*.*30)

*−*

Food engineer 0*.*35 (0*.*31)

*−*

Accountant 0*.*46 (0*.*31)

*—* ∗

Up to 3 months 0*.*98 (0*.*30)

*—* ∗

Up to 6 months 1*.*62 (0*.*30)

*—* ∗

Up to 1 year 2*.*93 (0*.*30)

*—* ∗

Effort: Looking, 1-2 appl./week 7*.*98 (0*.*31)

Effort: Looking, 3-4 appl./week 8*.*80 (0*.*31)^∗^

Effort: Looking, 5-6 appl./week 9*.*88 (0*.*30)^∗^ Resp. controls

Resp. age 0*.*10 (0*.*01)^∗^

*−*

Resp. not native born 2*.*16 (0*.*74)^∗^ Upper sec./post-sec. non-tertiary 0*.*06 (0*.*61) Tertiary (BA, MA, Professional, PhD) 1*.*05 (0*.*66) Male 1*.*00 (0*.*42)^∗^

*−*

Available savings 0*.*74 (0*.*12)^∗^

*−*

Unempl. 0*.*12 (0*.*85)

Country dummies

Germany *−*2*.*08 (0*.*72)^∗^

Denmark *−*5*.*72 (0*.*86)^∗^

Sweden *−*4*.*82 (0*.*84)^∗^

United Kingdom *−*14*.*42 (0*.*85)^∗^

United States *−*9*.*32 (0*.*74)^∗^

AIC 253881*.*73

BIC 254211*.*61

Log Likelihood 126900*.*87

*−*

Observations 28195

Respondents 9399

Var: Resp. intercept 315*.*13

Var: Residual 294*.*47

∗*p <* 0*.*05

Table S-11: Baseline model (pooled)

Nigerian *−*2*.*65 (0*.*34)^∗^ *−*2*.*74 (0*.*36)^∗^ *−*2*.*65 (0*.*34)^∗^ *−*2*.*74 (0*.*35)^∗^

|  | Model 1 | Model 2 | Model 3 | Model 4 |
| --- | --- | --- | --- | --- |
| Intercept | 65*.*51 (1*.*29)^∗^ | 58*.*20 (1*.*29)^∗^ | 55*.*69 (1*.*23)^∗^ | 65*.*13 (1*.*40)^∗^ |
| Vignette attributes  Female | 0*.*94 (0*.*21)^∗^ | 0*.*89 (0*.*23)^∗^ | 0*.*94 (0*.*21)^∗^ | 0*.*89 (0*.*23)^∗^ |
| Age: 40 years | 0*.*54 (0*.*26)^∗^ | 0*.*54 (0*.*27)^∗^ | 0*.*55 (0*.*26)^∗^ | 0*.*54 (0*.*27)^∗^ |
| Age: 55 years | 1*.*29 (0*.*26)^∗^ | 1*.*27 (0*.*28)^∗^ | 1*.*29 (0*.*26)^∗^ | 1*.*27 (0*.*28)^∗^ |
| Commute too long | 5*.*74 (0*.*31)^∗^ | 5*.*79 (0*.*32)^∗^ | 5*.*73 (0*.*31)^∗^ | 5*.*79 (0*.*32)^∗^ |
| Excessive overtime | 5*.*29 (0*.*30)^∗^ | 5*.*15 (0*.*32)^∗^ | 5*.*29 (0*.*30)^∗^ | 5*.*15 (0*.*32)^∗^ |
| Closure due to COVID | 15*.*23 (0*.*30)^∗^ | 15*.*36 (0*.*32)^∗^ | 15*.*23 (0*.*30)^∗^ | 15*.*35 (0*.*32)^∗^ |
| Tax/Contr: 2 years | 1*.*04 (0*.*30)^∗^ | 1*.*07 (0*.*32)^∗^ | 1*.*05 (0*.*30)^∗^ | 1*.*07 (0*.*32)^∗^ |
| Tax/Contr: 4 years | 1*.*46 (0*.*30)^∗^ | 1*.*47 (0*.*32)^∗^ | 1*.*46 (0*.*30)^∗^ | 1*.*47 (0*.*32)^∗^ |
| Tax/Contr: 8 years | 2*.*89 (0*.*30)^∗^ | 2*.*94 (0*.*32)^∗^ | 2*.*88 (0*.*30)^∗^ | 2*.*95 (0*.*32)^∗^ |
| Residency: 10 years  Residency: 5 years  Residency: 2 years | *−*1*.*41 (0*.*30)^∗^  *−*2*.*23 (0*.*30)^∗^  *−*3*.*43 (0*.*30)^∗^ | *−*1*.*49 (0*.*32)^∗^  *−*2*.*34 (0*.*32)^∗^  *−*3*.*40 (0*.*32)^∗^ | *−*1*.*42 (0*.*30)^∗^  *−*2*.*23 (0*.*30)^∗^  *−*3*.*44 (0*.*30)^∗^ | *−*1*.*47 (0*.*32)^∗^  *−*2*.*34 (0*.*32)^∗^  *−*3*.*39 (0*.*32)^∗^ |
| Neighboring  Ukrainian Afghan | *−*2*.*05 (0*.*35)^∗^  *−*2*.*31 (0*.*34)^∗^  *−*3*.*67 (0*.*35)^∗^ | *−*2*.*25 (0*.*36)^∗^  *−*2*.*50 (0*.*36)^∗^  *−*3*.*78 (0*.*37)^∗^ | *−*2*.*05 (0*.*35)^∗^  *−*2*.*29 (0*.*34)^∗^  *−*3*.*66 (0*.*35)^∗^ | *−*2*.*25 (0*.*36)^∗^  *−*2*.*53 (0*.*36)^∗^  *−*3*.*79 (0*.*37)^∗^ |

Lab technician *−*0*.*33 (0*.*30) *−*0*.*30 (0*.*32) *−*0*.*33 (0*.*30) *−*0*.*30 (0*.*32)

Food engineer *−*0*.*36 (0*.*31) *−*0*.*37 (0*.*32) *−*0*.*35 (0*.*31) *−*0*.*38 (0*.*32)

Accountant 0*.*48 (0*.*31)

*—* ∗

*—* ∗

*—* ∗

*—* ∗

Up to 3 months 0*.*99 (0*.*30)

*—* ∗

Up to 6 months 1*.*61 (0*.*30)

*—* ∗

Up to 1 year 2*.*95 (0*.*30)

*—* ∗

Effort: Looking, 1-2 appl./week 7*.*99 (0*.*31)

0*.*39 (0*.*32)

0*.*92 (0*.*32)

*—* ∗

1*.*68 (0*.*32)

*—* ∗

2*.*80 (0*.*32)

*—* ∗

8*.*15 (0*.*32)

0*.*46 (0*.*31)

0*.*98 (0*.*30)

*—* ∗

1*.*62 (0*.*30)

*—* ∗

2*.*94 (0*.*30)

*—* ∗

7*.*98 (0*.*31)

0*.*41 (0*.*32)

0*.*93 (0*.*32)

*—* ∗

1*.*66 (0*.*32)

*—* ∗

2*.*81 (0*.*32)

*—* ∗

8*.*16 (0*.*32)

Effort: Looking, 3-4 appl./week 8*.*80 (0*.*31)^∗^ 8*.*97 (0*.*33)^∗^ 8*.*80 (0*.*31)^∗^ 8*.*97 (0*.*33)^∗^

Effort: Looking, 5-6 appl./week 9*.*89 (0*.*30)^∗^ 10*.*03 (0*.*32)^∗^ 9*.*88 (0*.*30)^∗^ 10*.*04 (0*.*32)^∗^ Resp. controls

Resp. age 0*.*09 (0*.*01)^∗^ 0*.*09 (0*.*01)^∗^ 0*.*10 (0*.*01)^∗^ 0*.*08 (0*.*01)^∗^

*— − − −*

Resp. not native born 2*.*00 (0*.*73)^∗^ 2*.*37 (0*.*74)^∗^

Upper sec./post-sec. non-tertiary *−*0*.*31 (0*.*60) *−*0*.*45 (0*.*64) *−*0*.*10 (0*.*61) *−*0*.*76 (0*.*63)

Tertiary (BA, MA, Professional, PhD) 0*.*40 (0*.*65) 0*.*39 (0*.*69)

∗

0*.*69 (0*.*66)

∗

*−*0*.*27 (0*.*69)

Male *−*0*.*33 (0*.*42)

Available savings *−*0*.*59 (0*.*12)

*−*0*.*93 (0*.*45)

*−*0*.*71 (0*.*13)

∗

*−*0*.*95 (0*.*42)

*−*0*.*73 (0*.*12)

∗

*−*0*.*30 (0*.*44)

*−*0*.*57 (0*.*13)

∗

∗

Unempl. 0*.*21 (0*.*83) 0*.*29 (0*.*91) 0*.*02 (0*.*85) 0*.*76 (0*.*89)

*— − −*

Personality variables

SDO 2*.*26 (0*.*13)^∗^ 2*.*06 (0*.*14)^∗^

*— −*

Authoritarianism 1*.*55 (0*.*24)^∗^ 0*.*76 (0*.*26)^∗^

*— −*

Ethnocentrism 2*.*21 (0*.*22)^∗^ 1*.*60 (0*.*22)^∗^ Country dummies

*— −*

Germany *−*1*.*78 (0*.*71)^∗^ *−*2*.*44 (0*.*78)^∗^ *−*2*.*32 (0*.*72)^∗^ *−*2*.*29 (0*.*77)^∗^

Denmark *−*6*.*44 (0*.*85)^∗^ *−*5*.*68 (0*.*92)^∗^ *−*5*.*81 (0*.*86)^∗^ *−*6*.*52 (0*.*91)^∗^

Sweden *−*6*.*51 (0*.*84)^∗^ *−*5*.*28 (0*.*92)^∗^ *−*4*.*78 (0*.*84)^∗^ *−*6*.*89 (0*.*92)^∗^

United Kingdom *−*15*.*63 (0*.*84)^∗^ *−*15*.*87 (0*.*94)^∗^ *−*14*.*27 (0*.*85)^∗^ *−*16*.*69 (0*.*93)^∗^

United States *−*10*.*77 (0*.*73)^∗^ *−*10*.*61 (0*.*81)^∗^ *−*8*.*66 (0*.*74)^∗^ *−*11*.*34 (0*.*81)^∗^

AIC 253577*.*58 230022*.*61 253844*.*38 229781*.*43

BIC 253915*.*71 230348*.*52 254182*.*50 230123*.*63

Log Likelihood 126747*.*79 114971*.*31 126881*.*19 114848*.*72

*— − − −*

Observations 28195 25531 28195 25531

Respondents 9399 8511 9399 8511

Var: Resp. intercept 301*.*81 313*.*16 313*.*40 301*.*39

Var: Residual 294*.*47 297*.*03 294*.*47 297*.*03

∗ *p <* 0*.*05

Table S-12: Main effects of personality variables (pooled)

## Interactive models (immigrant vs. non-immigrant)

Starts overleaf.

Residency: 5 years *−*2*.*26 (0*.*30)^∗^ *−*2*.*27 (0*.*30)^∗^ *−*2*.*37 (0*.*32)^∗^

|  | Model 1 | Model 2 | Model 3 |
| --- | --- | --- | --- |
| Intercept | 63*.*64 (1*.*38)^∗^ | 55*.*78 (1*.*24)^∗^ | 57*.*74 (1*.*29)^∗^ |
| Vignette attributes  Female | 0*.*93 (0*.*21)^∗^ | 0*.*93 (0*.*21)^∗^ | 0*.*89 (0*.*23)^∗^ |
| Age: 40 years | 0*.*53 (0*.*26)^∗^ | 0*.*53 (0*.*26)^∗^ | 0*.*54 (0*.*27)^∗^ |
| Age: 55 years | 1*.*30 (0*.*26)^∗^ | 1*.*29 (0*.*26)^∗^ | 1*.*28 (0*.*27)^∗^ |
| Commute too long | 5*.*72 (0*.*31)^∗^ | 5*.*72 (0*.*31)^∗^ | 5*.*79 (0*.*32)^∗^ |
| Excessive overtime | 5*.*32 (0*.*30)^∗^ | 5*.*31 (0*.*30)^∗^ | 5*.*18 (0*.*32)^∗^ |
| Closure due to COVID | 15*.*22 (0*.*30)^∗^ | 15*.*23 (0*.*30)^∗^ | 15*.*36 (0*.*32)^∗^ |
| Tax/Contr: 2 years | 1*.*07 (0*.*30)^∗^ | 1*.*07 (0*.*30)^∗^ | 1*.*10 (0*.*32)^∗^ |
| Tax/Contr: 4 years | 1*.*49 (0*.*30)^∗^ | 1*.*48 (0*.*30)^∗^ | 1*.*50 (0*.*32)^∗^ |
| Tax/Contr: 8 years | 2*.*94 (0*.*30)^∗^ | 2*.*93 (0*.*30)^∗^ | 3*.*00 (0*.*32)^∗^ |
| Residency: 10 years | *−*1*.*44 (0*.*30)^∗^ | *−*1*.*45 (0*.*30)^∗^ | *−*1*.*53 (0*.*32)^∗^ |

Residency: 2 years *−*3*.*46 (0*.*30)^∗^ *−*3*.*47 (0*.*30)^∗^ *−*3*.*45 (0*.*32)^∗^

Lab technician *−*0*.*35 (0*.*30) *−*0*.*35 (0*.*30) *−*0*.*32 (0*.*32)

Food engineer *−*0*.*41 (0*.*31) *−*0*.*39 (0*.*31) *−*0*.*42 (0*.*32)

Accountant 0*.*54 (0*.*31)

*—* ∗

*—* ∗

*—* ∗

Up to 3 months 0*.*98 (0*.*30)

*—* ∗

Up to 6 months 1*.*61 (0*.*30)

*—* ∗

Up to 1 year 2*.*93 (0*.*30)

*—* ∗

Effort: Looking, 1-2 appl./week 8*.*01 (0*.*31)

0*.*52 (0*.*31)

0*.*97 (0*.*30)

*—* ∗

1*.*63 (0*.*30)

*—* ∗

2*.*92 (0*.*30)

*—* ∗

8*.*00 (0*.*31)

0*.*45 (0*.*32)

0*.*90 (0*.*32)

*—* ∗

1*.*67 (0*.*32)

*—* ∗

2*.*77 (0*.*32)

*—* ∗

8*.*16 (0*.*32)

Effort: Looking, 3-4 appl./week 8*.*81 (0*.*31)^∗^ 8*.*81 (0*.*31)^∗^ 8*.*97 (0*.*33)^∗^

Effort: Looking, 5-6 appl./week 9*.*89 (0*.*30)^∗^ 9*.*88 (0*.*30)^∗^ 10*.*04 (0*.*32)^∗^ Resp. controls

Resp. age 0*.*09 (0*.*01)^∗^ 0*.*10 (0*.*01)^∗^ 0*.*09 (0*.*01)^∗^

*— − −*

Resp. not native born 1*.*97 (0*.*73)^∗^ 2*.*32 (0*.*74)^∗^

Upper sec./post-sec. non-tertiary 0*.*33 (0*.*60) 0*.*12 (0*.*61) 0*.*46 (0*.*64)

*— − −*

Tertiary (BA, MA, Professional, PhD) 0*.*37 (0*.*65) 0*.*66 (0*.*66) 0*.*37 (0*.*69)

Male 0*.*31 (0*.*42) 0*.*92 (0*.*42)^∗^ 0*.*90 (0*.*45)^∗^

*— − −*

Available savings 0*.*59 (0*.*12)^∗^ 0*.*73 (0*.*12)^∗^ 0*.*72 (0*.*13)^∗^

*— − −*

Unempl. 0*.*20 (0*.*83) 0*.*03 (0*.*85) 0*.*30 (0*.*91)

*— −*

Personality variables

SDO 1*.*79 (0*.*18)^∗^

*−*

Authoritarianism 1*.*46 (0*.*33)^∗^

*−*

Ethnocentrism 1*.*05 (0*.*29)^∗^

*−*

Interaction

Immigrant *−*0*.*25 (0*.*69) *−*2*.*73 (0*.*35)^∗^ *−*2*.*24 (0*.*30)^∗^

Immigrant x SDO *−*0*.*60 (0*.*16)^∗^

Immigrant x author. 0*.*11 (0*.*30)

*−*

Immigrant x ethnoc. 1*.*49 (0*.*26)^∗^

*−*

Country dummies

Germany *−*1*.*79 (0*.*71)^∗^ *−*2*.*34 (0*.*72)^∗^ *−*2*.*44 (0*.*78)^∗^

Denmark *−*6*.*38 (0*.*85)^∗^ *−*5*.*76 (0*.*86)^∗^ *−*5*.*63 (0*.*92)^∗^

Sweden *−*6*.*52 (0*.*84)^∗^ *−*4*.*79 (0*.*84)^∗^ *−*5*.*26 (0*.*92)^∗^

United Kingdom *−*15*.*64 (0*.*84)^∗^ *−*14*.*28 (0*.*85)^∗^ *−*15*.*85 (0*.*94)^∗^

United States *−*10*.*77 (0*.*73)^∗^ *−*8*.*67 (0*.*74)^∗^ *−*10*.*59 (0*.*81)^∗^

AIC 253578*.*85 253854*.*71 230000*.*39

BIC 253916*.*97 254192*.*84 230326*.*29

Log Likelihood 126748*.*42 126886*.*36 114960*.*19

*— − −*

Observations 28195 28195 25531

Respondents 9399 9399 8511

Var: Resp. intercept 279*.*84 286*.*51 289*.*78

Var: Immigrant 1*.*68 2*.*78 3*.*09

Cov: intercept-immigrant 13*.*42 16*.*13 13*.*77

Var: Residual 294*.*16 294*.*14 296*.*10

∗ *p <* 0*.*05

Table S-13: Main interactive models (pooled)

Residency: 5 years *−*2*.*37 (0*.*32)^∗^ *−*2*.*37 (0*.*32)^∗^

|  | Model 1 | Model 2 |
| --- | --- | --- |
| Intercept | 63*.*39 (1*.*50)^∗^ | 64*.*62 (1*.*40)^∗^ |
| Vignette attributes  Female | 0*.*89 (0*.*23)^∗^ | 0*.*89 (0*.*23)^∗^ |
| Age: 40 years | 0*.*53 (0*.*27)^∗^ | 0*.*54 (0*.*27)^∗^ |
| Age: 55 years | 1*.*29 (0*.*27)^∗^ | 1*.*28 (0*.*27)^∗^ |
| Commute too long | 5*.*78 (0*.*32)^∗^ | 5*.*80 (0*.*32)^∗^ |
| Excessive overtime | 5*.*17 (0*.*32)^∗^ | 5*.*17 (0*.*32)^∗^ |
| Closure due to COVID | 15*.*35 (0*.*32)^∗^ | 15*.*36 (0*.*32)^∗^ |
| Tax/Contr: 2 years | 1*.*09 (0*.*32)^∗^ | 1*.*10 (0*.*32)^∗^ |
| Tax/Contr: 4 years | 1*.*50 (0*.*32)^∗^ | 1*.*50 (0*.*32)^∗^ |
| Tax/Contr: 8 years | 3*.*00 (0*.*32)^∗^ | 3*.*02 (0*.*32)^∗^ |
| Residency: 10 years | *−*1*.*50 (0*.*32)^∗^ | *−*1*.*51 (0*.*32)^∗^ |

Residency: 2 years *−*3*.*43 (0*.*32)^∗^ *−*3*.*44 (0*.*32)^∗^

Lab technician *−*0*.*31 (0*.*32) *−*0*.*32 (0*.*32)

Food engineer *−*0*.*42 (0*.*32) *−*0*.*43 (0*.*32)

Accountant 0*.*46 (0*.*32)

*—* ∗

*—* ∗

Up to 3 months 0*.*92 (0*.*32)

*—* ∗

Up to 6 months 1*.*67 (0*.*32)

*—* ∗

Up to 1 year 2*.*81 (0*.*32)

*—* ∗

Effort: Looking, 1-2 appl./week 8*.*18 (0*.*32)

0*.*46 (0*.*32)

0*.*92 (0*.*32)

*—* ∗

1*.*66 (0*.*32)

*—* ∗

2*.*79 (0*.*32)

*—* ∗

8*.*17 (0*.*32)

Effort: Looking, 3-4 appl./week 8*.*98 (0*.*33)^∗^ 8*.*97 (0*.*33)^∗^

Effort: Looking, 5-6 appl./week 10*.*04 (0*.*32)^∗^ 10*.*04 (0*.*32)^∗^ Resp. controls

Resp. age *−*0*.*08 (0*.*01)^∗^ *−*0*.*08 (0*.*01)^∗^

Upper sec./post-sec. non-tertiary *−*0*.*77 (0*.*63) *−*0*.*77 (0*.*63)

Tertiary (BA, MA, Professional, PhD) *−*0*.*29 (0*.*69) *−*0*.*28 (0*.*69)

Male *−*0*.*28 (0*.*44)

Available savings *−*0*.*58 (0*.*13)

*−*0*.*28 (0*.*44)

*−*0*.*58 (0*.*13)

∗

∗

Unempl. 0*.*75 (0*.*89) 0*.*77 (0*.*89)

*— −*

Personality variables

SDO 1*.*63 (0*.*19)^∗^ 2*.*05 (0*.*14)^∗^

*— −*

Authoritarianism 0*.*77 (0*.*26)^∗^ 0*.*77 (0*.*26)^∗^

*— −*

Ethnocentrism 1*.*57 (0*.*22)^∗^ 0*.*45 (0*.*29) Interactions

*— −*

Immigrant 0*.*63 (0*.*73) 2*.*25 (0*.*30)^∗^

*— −*

Immigrant x SDO 0*.*55 (0*.*17)^∗^

*−*

Immigrant x ethnoc. 1*.*48 (0*.*26)^∗^

*−*

Country dummies

Germany *−*2*.*28 (0*.*77)^∗^ *−*2*.*29 (0*.*77)^∗^

Denmark *−*6*.*46 (0*.*91)^∗^ *−*6*.*49 (0*.*91)^∗^

Sweden *−*6*.*87 (0*.*92)^∗^ *−*6*.*86 (0*.*92)^∗^

United Kingdom *−*16*.*66 (0*.*93)^∗^ *−*16*.*66 (0*.*93)^∗^

United States *−*11*.*31 (0*.*81)^∗^ *−*11*.*31 (0*.*81)^∗^

| AIC | 229783*.*94 | 229761*.*47 |
| --- | --- | --- |
| BIC | 230126*.*14 | 230103*.*67 |
| Log Likelihood | *−*114849*.*97 | *−*114838*.*73 |

Observations 25531 25531

Respondents 8511 8511

Var: Resp. intercept 282*.*69 281*.*36

Var: Immigrant 4*.*62 3*.*09

Cov: intercept-immigrant 10*.*28 11*.*65

Var: Residual 296*.*09 296*.*10

∗ *p <* 0*.*05

Table S-14: Interactive models – robustness checks (pooled)

*−*1*.*83 (1*.*07)

|  | Model 1 | Model 2 |
| --- | --- | --- |
| Intercept | 58*.*51 (5*.*45)^∗^ | 58*.*65 (5*.*48)^∗^ |
| Vignette attributes |  |  |
| Female | 1*.*07 (0*.*66) | 1*.*06 (0*.*66) |
| Age: 40 years | 1*.*30 (0*.*79) | 1*.*41 (0*.*79) |
| Age: 55 years | 2*.*19 (0*.*80)^∗^ | 2*.*34 (0*.*80)^∗^ |
| Commute too long | 4*.*63 (0*.*94)^∗^ | 4*.*62 (0*.*94)^∗^ |
| Excessive overtime | 2*.*78 (0*.*93)^∗^ | 2*.*73 (0*.*93)^∗^ |
| Closure due to COVID | 15*.*84 (0*.*92)^∗^ | 15*.*83 (0*.*92)^∗^ |
| Tax/Contr: 2 years | 1*.*14 (0*.*93) | 1*.*13 (0*.*93) |
| Tax/Contr: 4 years | 1*.*37 (0*.*92) | 1*.*33 (0*.*92) |
| Tax/Contr: 8 years | 2*.*64 (0*.*94)^∗^ | 2*.*56 (0*.*93)^∗^ |
| Residency: 10 years  Residency: 5 years  Residency: 2 years Neighboring | *−*2*.*14 (0*.*93)^∗^  *−*2*.*68 (0*.*95)^∗^  *−*2*.*61 (0*.*92)^∗^ | *−*2*.*12 (0*.*93)^∗^  *−*2*.*68 (0*.*94)^∗^  *−*2*.*58 (0*.*92)^∗^ |
| Ukrainian  Afghan Nigerian  Lab technician | *−*0*.*55 (1*.*04)  0*.*01 (1*.*04)  0*.*16 (0*.*93) | 0*.*11 (0*.*93) |
| Food engineer | *−*0*.*23 (0*.*93) | *−*0*.*23 (0*.*93) |

*−*1*.*21 (1*.*06)

Accountant *−*1*.*15 (0*.*94) *−*1*.*14 (0*.*94)

Up to 3 months 0*.*67 (0*.*95)

*—* ∗

*—* ∗

Up to 6 months 2*.*05 (0*.*93)

*—* ∗

Up to 1 year 3*.*10 (0*.*93)

*—* ∗

Effort: Looking, 1-2 appl./week 9*.*54 (0*.*93)

0*.*69 (0*.*95)

2*.*09 (0*.*93)

*—* ∗

3*.*11 (0*.*93)

*—* ∗

9*.*51 (0*.*93)

Effort: Looking, 3-4 appl./week 9*.*40 (0*.*95)^∗^ 9*.*41 (0*.*95)^∗^

Effort: Looking, 5-6 appl./week 11*.*56 (0*.*92)^∗^ 11*.*56 (0*.*92)^∗^ Resp. controls

Resp. age 0*.*12 (0*.*04)^∗^ 0*.*12 (0*.*04)^∗^

*— −*

Resp. not US-born 2*.*34 (2*.*78) 2*.*35 (2*.*78)

Upper sec./post-sec. non-tertiary 10*.*31 (4*.*84)^∗^ 10*.*33 (4*.*84)^∗^ Tertiary (BA, MA, Professional, PhD) 9*.*33 (4*.*86) 9*.*31 (4*.*87)

*— −*

*— −*

Male 0*.*01 (1*.*30) 0*.*01 (1*.*30)

Black/Afr.Amer. 7*.*68 (2*.*54)^∗^ 7*.*72 (2*.*54)^∗^

Asian/Pac.Isl. 0*.*61 (3*.*20)

*—* ∗

Hispanic 8*.*42 (3*.*86)

Other *−*7*.*02 (3*.*78)

Available savings *−*1*.*23 (0*.*36)

0*.*59 (3*.*20)

8*.*48 (3*.*86)

*—* ∗

*−*7*.*01 (3*.*78)

∗

∗

*−*1*.*23 (0*.*36)

Unempl. 0*.*80 (1*.*88) 0*.*78 (1*.*88)

*— −*

Interaction

Immigrant 1*.*13 (1*.*15)

*−*

IAT D-score 0*.*80 (1*.*52) 1*.*34 (2*.*23)

*— −*

Immigrant x IAT D-score 0*.*67 (2*.*01)

AIC 30496*.*74 30501*.*87

BIC 30741*.*49 30746*.*63

Log Likelihood 15208*.*37 15210*.*94

*— −*

Observations 3357 3357

Respondents 1119 1119

Var: Resp. intercept 322*.*76 327*.*92

Var: Residual 329*.*48 328*.*02

Var: Immigrant 7*.*25

Cov: intercept-immigrant *−*5*.*74

∗ *p <* 0*.*05

Table S-15: Models of effects of IAT *D*-scores (US)

Residency: 5 years *−*2*.*30 (0*.*77)^∗^ *−*2*.*40 (0*.*77)^∗^

|  | Model 1 | Model 2 |
| --- | --- | --- |
| Intercept | 52*.*96 (2*.*68)^∗^ | 52*.*94 (2*.*74)^∗^ |
| Vignette attributes  Female | 1*.*32 (0*.*55)^∗^ | 1*.*31 (0*.*55)^∗^ |
| Age: 40 years | 0*.*73 (0*.*66) | 0*.*72 (0*.*66) |
| Age: 55 years | 1*.*01 (0*.*67) | 1*.*00 (0*.*67) |
| Commute too long | 6*.*01 (0*.*80)^∗^ | 5*.*99 (0*.*80)^∗^ |
| Excessive overtime | 6*.*79 (0*.*78)^∗^ | 6*.*79 (0*.*78)^∗^ |
| Closure due to COVID | 16*.*83 (0*.*79)^∗^ | 16*.*83 (0*.*79)^∗^ |
| Tax/Contr: 2 years | 1*.*02 (0*.*77) | 1*.*02 (0*.*77) |
| Tax/Contr: 4 years | 1*.*46 (0*.*78) | 1*.*50 (0*.*78) |
| Tax/Contr: 8 years | 3*.*89 (0*.*77)^∗^ | 3*.*95 (0*.*77)^∗^ |
| Residency: 10 years | *−*2*.*36 (0*.*77)^∗^ | *−*2*.*38 (0*.*77)^∗^ |

Residency: 2 years *−*2*.*65 (0*.*78)^∗^ *−*2*.*74 (0*.*78)^∗^

Neighboring *−*1*.*38 (0*.*88)∗

Ukrainian *−*2*.*50 (0*.*86)∗

Afghan *−*3*.*55 (0*.*89)∗

Nigerian *−*2*.*07 (0*.*87)

Lab technician 0*.*23 (0*.*78) 0*.*19 (0*.*78)

Food engineer 0*.*14 (0*.*79) 0*.*04 (0*.*79)

Accountant 0*.*08 (0*.*78) 0*.*01 (0*.*78)

Up to 3 months 1*.*23 (0*.*78)

*−*

Up to 6 months 1*.*68 (0*.*78)

*−*

Up to 1 year 1*.*14 (0*.*78)

*—* ∗

Effort: Looking, 1-2 appl./week 7*.*59 (0*.*79)

1*.*18 (0*.*78)

1*.*70 (0*.*78)

∗

∗

*−*

*−*

1*.*14 (0*.*77)

*—* ∗

7*.*61 (0*.*79)

Effort: Looking, 3-4 appl./week 8*.*34 (0*.*79)^∗^ 8*.*33 (0*.*79)^∗^

Effort: Looking, 5-6 appl./week 9*.*02 (0*.*78)^∗^ 9*.*07 (0*.*78)^∗^ Resp. controls

Resp. age 0*.*11 (0*.*03)^∗^ 0*.*11 (0*.*03)^∗^

*— −*

Resp. not DE-born 0*.*52 (2*.*38) 0*.*40 (2*.*39)

Upper sec./post-sec. non-tertiary 0*.*72 (1*.*30) 0*.*64 (1*.*30)

Tertiary (BA, MA, Professional, PhD) 0*.*65 (1*.*52) 0*.*75 (1*.*52)

*— −*

Male 1*.*15 (1*.*04) 1*.*14 (1*.*04)

*— −*

Available savings 0*.*40 (0*.*31) 0*.*42 (0*.*31)

*— −*

Unempl. 3*.*79 (2*.*79) 3*.*69 (2*.*79)

Interaction

Immigrant 2*.*15 (1*.*01)^∗^

*−*

IAT D-score 0*.*39 (1*.*26) 0*.*66 (1*.*75)

Immigrant x IAT D-score *−*0*.*38 (1*.*63)

AIC 33085*.*03 33089*.*62

BIC 33309*.*26 33313*.*86

Log Likelihood 16506*.*51 16508*.*81

*— −*

Observations 3747 3747

Respondents 1249 1249

Var: Resp. intercept 240*.*84 221*.*04

Var: Residual 255*.*96 253*.*07

Var: Immigrant 14*.*29

Cov: intercept-immigrant 7*.*73

∗*p <* 0*.*05

Table S-16: Models of effects of IAT *D*-scores (DE)

# Alternative codings of immigrant attribute

## Light- vs. dark-skinned

(Starts overleaf.)

Food engineer *−*0*.*39 (0*.*31) *−*0*.*42 (0*.*32) *−*0*.*39 (0*.*31) *−*0*.*41 (0*.*32) *−*0*.*39 (0*.*31) *−*0*.*42 (0*.*32)

|  | Model 1 | Model 2 | Model 3 | Model 4 | Model 5 | Model 6 |
| --- | --- | --- | --- | --- | --- | --- |
| Intercept | 55*.*46 (1*.*20)^∗^ | 63*.*31 (1*.*39)^∗^ | 62*.*93 (1*.*29)^∗^ | 56*.*20 (1*.*27)^∗^ | 54*.*31 (1*.*22)^∗^ | 63*.*49 (1*.*39)^∗^ |
| Vignette attributes  Female | 0*.*94 (0*.*21)^∗^ | 0*.*90 (0*.*23)^∗^ | 0*.*95 (0*.*21)^∗^ | 0*.*90 (0*.*23)^∗^ | 0*.*95 (0*.*21)^∗^ | 0*.*90 (0*.*23)^∗^ |
| Age: 40 years | 0*.*66 (0*.*26)^∗^ | 0*.*66 (0*.*27)^∗^ | 0*.*67 (0*.*26)^∗^ | 0*.*67 (0*.*27)^∗^ | 0*.*67 (0*.*26)^∗^ | 0*.*67 (0*.*27)^∗^ |
| Age: 55 years | 1*.*43 (0*.*26)^∗^ | 1*.*42 (0*.*27)^∗^ | 1*.*43 (0*.*26)^∗^ | 1*.*42 (0*.*27)^∗^ | 1*.*43 (0*.*26)^∗^ | 1*.*42 (0*.*27)^∗^ |
| Commute too long | 5*.*73 (0*.*31)^∗^ | 5*.*78 (0*.*32)^∗^ | 5*.*74 (0*.*31)^∗^ | 5*.*78 (0*.*32)^∗^ | 5*.*73 (0*.*31)^∗^ | 5*.*77 (0*.*32)^∗^ |
| Excessive overtime | 5*.*28 (0*.*31)^∗^ | 5*.*13 (0*.*32)^∗^ | 5*.*28 (0*.*30)^∗^ | 5*.*13 (0*.*32)^∗^ | 5*.*28 (0*.*31)^∗^ | 5*.*12 (0*.*32)^∗^ |
| Closure due to COVID | 15*.*18 (0*.*30)^∗^ | 15*.*29 (0*.*32)^∗^ | 15*.*17 (0*.*30)^∗^ | 15*.*30 (0*.*32)^∗^ | 15*.*18 (0*.*30)^∗^ | 15*.*29 (0*.*32)^∗^ |
| Tax/Contr: 2 years | 1*.*03 (0*.*30)^∗^ | 1*.*06 (0*.*32)^∗^ | 1*.*02 (0*.*30)^∗^ | 1*.*04 (0*.*32)^∗^ | 1*.*04 (0*.*30)^∗^ | 1*.*07 (0*.*32)^∗^ |
| Tax/Contr: 4 years | 1*.*43 (0*.*30)^∗^ | 1*.*44 (0*.*32)^∗^ | 1*.*43 (0*.*30)^∗^ | 1*.*41 (0*.*32)^∗^ | 1*.*44 (0*.*30)^∗^ | 1*.*44 (0*.*32)^∗^ |
| Tax/Contr: 8 years | 2*.*88 (0*.*30)^∗^ | 2*.*95 (0*.*32)^∗^ | 2*.*88 (0*.*30)^∗^ | 2*.*94 (0*.*32)^∗^ | 2*.*88 (0*.*30)^∗^ | 2*.*95 (0*.*32)^∗^ |
| Residency: 10 years  Residency: 5 years | *−*1*.*41 (0*.*30)^∗^  *−*2*.*23 (0*.*30)^∗^ | *−*1*.*45 (0*.*32)^∗^  *−*2*.*32 (0*.*32)^∗^ | *−*1*.*41 (0*.*30)^∗^  *−*2*.*22 (0*.*30)^∗^ | *−*1*.*44 (0*.*32)^∗^  *−*2*.*31 (0*.*32)^∗^ | *−*1*.*41 (0*.*30)^∗^  *−*2*.*22 (0*.*30)^∗^ | *−*1*.*45 (0*.*32)^∗^  *−*2*.*32 (0*.*32)^∗^ |
| Residency: 2 years  Lab technician | *−*3*.*40 (0*.*30)^∗^  *−*0*.*34 (0*.*30) | *−*3*.*34 (0*.*32)^∗^  *−*0*.*31 (0*.*32) | *−*3*.*39 (0*.*30)^∗^  *−*0*.*33 (0*.*30) | *−*3*.*33 (0*.*32)^∗^  *−*0*.*31 (0*.*32) | *−*3*.*39 (0*.*30)^∗^  *−*0*.*34 (0*.*30) | *−*3*.*34 (0*.*32)^∗^  *−*0*.*30 (0*.*32) |

Accountant 0*.*44 (0*.*31)

*—* ∗

*—* ∗

*—* ∗

*—* ∗

*—* ∗

*—* ∗

Up to 3 months 0*.*97 (0*.*30)

*—* ∗

Up to 6 months 1*.*62 (0*.*30)

*—* ∗

Up to 1 year 2*.*92 (0*.*30)

*—* ∗

Effort: Looking, 1-2 appl./week 7*.*99 (0*.*31)

0*.*38 (0*.*32)

0*.*92 (0*.*32)

*—* ∗

1*.*66 (0*.*32)

*—* ∗

2*.*80 (0*.*32)

*—* ∗

8*.*17 (0*.*32)

0*.*46 (0*.*31)

0*.*97 (0*.*30)

*—* ∗

1*.*62 (0*.*30)

*—* ∗

2*.*93 (0*.*30)

*—* ∗

8*.*01 (0*.*31)

0*.*35 (0*.*32)

0*.*89 (0*.*32)

*—* ∗

1*.*66 (0*.*32)

*—* ∗

2*.*77 (0*.*32)

*—* ∗

8*.*18 (0*.*32)

0*.*44 (0*.*31)

0*.*97 (0*.*30)

*—* ∗

1*.*63 (0*.*30)

*—* ∗

2*.*92 (0*.*30)

*—* ∗

7*.*99 (0*.*31)

0*.*38 (0*.*32)

0*.*92 (0*.*32)

*—* ∗

1*.*67 (0*.*32)

*—* ∗

2*.*80 (0*.*32)

*—* ∗

8*.*17 (0*.*32)

Effort: Looking, 3-4 appl./week 8*.*85 (0*.*31)^∗^ 9*.*02 (0*.*33)^∗^ 8*.*87 (0*.*31)^∗^ 9*.*05 (0*.*33)^∗^ 8*.*86 (0*.*31)^∗^ 9*.*03 (0*.*33)^∗^

Effort: Looking, 5-6 appl./week 9*.*93 (0*.*30)^∗^ 10*.*10 (0*.*32)^∗^ 9*.*93 (0*.*30)^∗^ 10*.*12 (0*.*32)^∗^ 9*.*94 (0*.*30)^∗^ 10*.*11 (0*.*32)^∗^

Resp. controls

Resp. age 0*.*10 (0*.*01)^∗^ 0*.*08 (0*.*01)^∗^ 0*.*09 (0*.*01)^∗^ 0*.*09 (0*.*01)^∗^ 0*.*10 (0*.*01)^∗^ 0*.*08 (0*.*01)^∗^

*— − − − − −*

Resp. not native born 2*.*19 (0*.*74)^∗^ 2*.*03 (0*.*73)^∗^ 2*.*39 (0*.*74)^∗^

Upper sec./post-sec. non-tertiary 0*.*07 (0*.*61) *−*0*.*74 (0*.*63) *−*0*.*31 (0*.*60) *−*0*.*44 (0*.*63) *−*0*.*09 (0*.*61) *−*0*.*73 (0*.*63)

Tertiary (BA, MA, Professional, PhD) 1*.*01 (0*.*66)

∗

*−*0*.*27 (0*.*69) 0*.*36 (0*.*65) 0*.*35 (0*.*69)∗

0*.*65 (0*.*66)

∗

*−*0*.*27 (0*.*69)

Male *−*0*.*97 (0*.*42)

Available savings *−*0*.*74 (0*.*12)

*−*0*.*28 (0*.*44)

*−*0*.*58 (0*.*13)

∗

*−*0*.*31 (0*.*42)

*−*0*.*59 (0*.*12)

∗

*−*0*.*88 (0*.*45)

*−*0*.*72 (0*.*13)

∗

*−*0*.*91 (0*.*42)

*−*0*.*73 (0*.*12)

∗

*−*0*.*28 (0*.*44)

*−*0*.*58 (0*.*13)

∗

∗

Unempl. 0*.*12 (0*.*85) *−*0*.*75 (0*.*89)∗

*−*0*.*20 (0*.*83)∗

*−*0*.*29 (0*.*91) 0*.*02 (0*.*85) *−*0*.*75 (0*.*89)∗

SDO *−*2*.*04 (0*.*14)∗

Authoritarianism *−*0*.*75 (0*.*26)∗

Ethnocentrism *−*1*.*55 (0*.*22)

Interactions

*−*2*.*00 (0*.*14)

*−*1*.*62 (0*.*23)^∗^

*−*1*.*31 (0*.*26)^∗^

*−*2*.*04 (0*.*14)

*−*0*.*50 (0*.*28)

∗

*−*1*.*55 (0*.*22)

Dark-skinned 1*.*71 (0*.*23)^∗^ 1*.*68 (0*.*24)^∗^ 0*.*91 (0*.*58) 1*.*14 (0*.*25)^∗^ 2*.*18 (0*.*30)^∗^ 2*.*22 (0*.*32)^∗^

*— − − − −*

Dark-skinned x SDO 0*.*66 (0*.*13)^∗^

*−*

Dark-skinned x authorit. 0*.*61 (0*.*25)^∗^ 0*.*70 (0*.*27)^∗^

*— −*

Dark-skinned x ethnoc. 1*.*54 (0*.*23)^∗^

*−*

Country dummies

Germany *−*2*.*11 (0*.*72)^∗^ *−*2*.*27 (0*.*77)^∗^ *−*1*.*81 (0*.*71)^∗^ *−*2*.*43 (0*.*78)^∗^ *−*2*.*35 (0*.*72)^∗^ *−*2*.*27 (0*.*77)^∗^

Denmark *−*5*.*60 (0*.*86)^∗^ *−*6*.*40 (0*.*91)^∗^ *−*6*.*33 (0*.*85)^∗^ *−*5*.*54 (0*.*92)^∗^ *−*5*.*69 (0*.*86)^∗^ *−*6*.*39 (0*.*91)^∗^

Sweden *−*4*.*81 (0*.*84)^∗^ *−*6*.*83 (0*.*91)^∗^ *−*6*.*50 (0*.*84)^∗^ *−*5*.*22 (0*.*92)^∗^ *−*4*.*77 (0*.*84)^∗^ *−*6*.*82 (0*.*92)^∗^

United Kingdom *−*14*.*42 (0*.*85)^∗^ *−*16*.*63 (0*.*93)^∗^ *−*15*.*63 (0*.*84)^∗^ *−*15*.*82 (0*.*94)^∗^ *−*14*.*27 (0*.*85)^∗^ *−*16*.*63 (0*.*93)^∗^

United States *−*9*.*34 (0*.*73)^∗^ *−*11*.*29 (0*.*81)^∗^ *−*10*.*77 (0*.*73)^∗^ *−*10*.*57 (0*.*81)^∗^ *−*8*.*69 (0*.*74)^∗^ *−*11*.*29 (0*.*81)^∗^

AIC 253919*.*61 229834*.*98 253603*.*10 230027*.*72 253880*.*60 229831*.*07

BIC 254241*.*24 230169*.*03 253941*.*22 230353*.*63 254218*.*72 230173*.*27

Log Likelihood 126920*.*81 114876*.*49 126760*.*55 114973*.*86 126899*.*30 114873*.*53

*— − − − − −*

Obervations 28195 25531 28195 25531 28195 25531

Respondents 9399 8511 9399 8511 9399 8511

Var: Resp. intercept 299*.*43 290*.*68 289*.*31 299*.*45 298*.*13 290*.*73

Var: Dark-skinned 4*.*48 6*.*89 4*.*53 3*.*58 3*.*95 6*.*58

Cov: intercept-Dark-skinned 18*.*97 12*.*43 15*.*08 16*.*75 18*.*57 12*.*51

Var: Residual 294*.*11 296*.*09 293*.*73 296*.*32 294*.*18 296*.*06

∗ *p <* 0*.*05

Table S-17: Interactive models – Alternative immigrant coding (light-vs. dark- skinned; pooled)

## WEIRD vs. Non-WEIRD

(Starts overleaf.)

Food engineer *−*0*.*39 (0*.*31) *−*0*.*41 (0*.*32) *−*0*.*40 (0*.*31) *−*0*.*40 (0*.*32) *−*0*.*39 (0*.*31) *−*0*.*41 (0*.*32)

|  | Model 1 | Model 2 | Model 3 | Model 4 | Model 5 | Model 6 |
| --- | --- | --- | --- | --- | --- | --- |
| Intercept | 56*.*04 (1*.*20)^∗^ | 63*.*96 (1*.*39)^∗^ | 62*.*80 (1*.*31)^∗^ | 56*.*63 (1*.*27)^∗^ | 54*.*98 (1*.*22)^∗^ | 64*.*23 (1*.*40)^∗^ |
| Vignette attributes  Female | 0*.*94 (0*.*21)^∗^ | 0*.*90 (0*.*23)^∗^ | 0*.*95 (0*.*21)^∗^ | 0*.*90 (0*.*23)^∗^ | 0*.*94 (0*.*21)^∗^ | 0*.*90 (0*.*23)^∗^ |
| Age: 40 years | 0*.*65 (0*.*26)^∗^ | 0*.*66 (0*.*27)^∗^ | 0*.*66 (0*.*26)^∗^ | 0*.*66 (0*.*27)^∗^ | 0*.*66 (0*.*26)^∗^ | 0*.*67 (0*.*27)^∗^ |
| Age: 55 years | 1*.*45 (0*.*26)^∗^ | 1*.*44 (0*.*27)^∗^ | 1*.*45 (0*.*26)^∗^ | 1*.*42 (0*.*27)^∗^ | 1*.*45 (0*.*26)^∗^ | 1*.*44 (0*.*27)^∗^ |
| Commute too long | 5*.*68 (0*.*31)^∗^ | 5*.*74 (0*.*32)^∗^ | 5*.*69 (0*.*31)^∗^ | 5*.*77 (0*.*32)^∗^ | 5*.*68 (0*.*31)^∗^ | 5*.*74 (0*.*32)^∗^ |
| Excessive overtime | 5*.*25 (0*.*31)^∗^ | 5*.*10 (0*.*32)^∗^ | 5*.*26 (0*.*30)^∗^ | 5*.*12 (0*.*32)^∗^ | 5*.*25 (0*.*31)^∗^ | 5*.*10 (0*.*32)^∗^ |
| Closure due to COVID | 15*.*17 (0*.*30)^∗^ | 15*.*28 (0*.*32)^∗^ | 15*.*14 (0*.*30)^∗^ | 15*.*30 (0*.*32)^∗^ | 15*.*16 (0*.*30)^∗^ | 15*.*27 (0*.*32)^∗^ |
| Tax/Contr: 2 years | 1*.*04 (0*.*30)^∗^ | 1*.*06 (0*.*32)^∗^ | 1*.*04 (0*.*30)^∗^ | 1*.*07 (0*.*32)^∗^ | 1*.*05 (0*.*30)^∗^ | 1*.*08 (0*.*32)^∗^ |
| Tax/Contr: 4 years | 1*.*44 (0*.*30)^∗^ | 1*.*45 (0*.*32)^∗^ | 1*.*45 (0*.*30)^∗^ | 1*.*47 (0*.*32)^∗^ | 1*.*45 (0*.*30)^∗^ | 1*.*46 (0*.*32)^∗^ |
| Tax/Contr: 8 years | 2*.*84 (0*.*30)^∗^ | 2*.*91 (0*.*32)^∗^ | 2*.*85 (0*.*30)^∗^ | 2*.*93 (0*.*32)^∗^ | 2*.*85 (0*.*30)^∗^ | 2*.*92 (0*.*32)^∗^ |
| Residency: 10 years  Residency: 5 years | *−*1*.*44 (0*.*30)^∗^  *−*2*.*23 (0*.*30)^∗^ | *−*1*.*46 (0*.*32)^∗^  *−*2*.*31 (0*.*32)^∗^ | *−*1*.*43 (0*.*30)^∗^  *−*2*.*21 (0*.*30)^∗^ | *−*1*.*47 (0*.*32)^∗^  *−*2*.*30 (0*.*32)^∗^ | *−*1*.*43 (0*.*30)^∗^  *−*2*.*22 (0*.*30)^∗^ | *−*1*.*46 (0*.*32)^∗^  *−*2*.*31 (0*.*32)^∗^ |
| Residency: 2 years  Lab technician | *−*3*.*41 (0*.*30)^∗^  *−*0*.*40 (0*.*30) | *−*3*.*35 (0*.*32)^∗^  *−*0*.*36 (0*.*32) | *−*3*.*40 (0*.*30)^∗^  *−*0*.*40 (0*.*30) | *−*3*.*36 (0*.*32)^∗^  *−*0*.*37 (0*.*32) | *−*3*.*40 (0*.*30)^∗^  *−*0*.*40 (0*.*30) | *−*3*.*35 (0*.*32)^∗^  *−*0*.*36 (0*.*32) |

Accountant 0*.*50 (0*.*31)

*—* ∗

*—* ∗

*—* ∗

*—* ∗

*—* ∗

*—* ∗

Up to 3 months 0*.*97 (0*.*30)

*—* ∗

Up to 6 months 1*.*62 (0*.*30)

*—* ∗

Up to 1 year 2*.*93 (0*.*30)

*—* ∗

Effort: Looking, 1-2 appl./week 7*.*98 (0*.*31)

0*.*43 (0*.*32)

0*.*92 (0*.*32)

*—* ∗

1*.*66 (0*.*32)

*—* ∗

2*.*81 (0*.*32)

*—* ∗

8*.*16 (0*.*32)

0*.*53 (0*.*31)

0*.*99 (0*.*30)

*—* ∗

1*.*62 (0*.*30)

*—* ∗

2*.*96 (0*.*30)

*—* ∗

8*.*00 (0*.*31)

0*.*43 (0*.*32)

0*.*90 (0*.*32)

*—* ∗

1*.*65 (0*.*32)

*—* ∗

2*.*78 (0*.*32)

*—* ∗

8*.*15 (0*.*32)

0*.*50 (0*.*31)

0*.*98 (0*.*30)

*—* ∗

1*.*62 (0*.*30)

*—* ∗

2*.*94 (0*.*30)

*—* ∗

7*.*98 (0*.*31)

0*.*44 (0*.*32)

0*.*93 (0*.*32)

*—* ∗

1*.*66 (0*.*32)

*—* ∗

2*.*82 (0*.*32)

*—* ∗

8*.*16 (0*.*32)

Effort: Looking, 3-4 appl./week 8*.*83 (0*.*31)^∗^ 9*.*00 (0*.*33)^∗^ 8*.*86 (0*.*31)^∗^ 9*.*02 (0*.*33)^∗^ 8*.*84 (0*.*31)^∗^ 9*.*01 (0*.*33)^∗^

Effort: Looking, 5-6 appl./week 9*.*93 (0*.*30)^∗^ 10*.*09 (0*.*32)^∗^ 9*.*94 (0*.*30)^∗^ 10*.*09 (0*.*32)^∗^ 9*.*94 (0*.*30)^∗^ 10*.*09 (0*.*32)^∗^

Resp. controls

Resp. age 0*.*10 (0*.*01)^∗^ 0*.*08 (0*.*01)^∗^ 0*.*09 (0*.*01)^∗^ 0*.*09 (0*.*01)^∗^ 0*.*10 (0*.*01)^∗^ 0*.*08 (0*.*01)^∗^

*— − − − − −*

Resp. not native born 2*.*13 (0*.*74)^∗^ 1*.*97 (0*.*73)^∗^ 2*.*34 (0*.*74)^∗^

Upper sec./post-sec. non-tertiary 0*.*01 (0*.*61) *−*0*.*78 (0*.*63) *−*0*.*35 (0*.*60) *−*0*.*49 (0*.*63) *−*0*.*15 (0*.*61) *−*0*.*78 (0*.*63)

Tertiary (BA, MA, Professional, PhD) 0*.*99 (0*.*66)

∗

*−*0*.*29 (0*.*69) 0*.*35 (0*.*65) 0*.*34 (0*.*69)∗

0*.*63 (0*.*66)

∗

*−*0*.*29 (0*.*69)

Male *−*0*.*98 (0*.*42)

Available savings *−*0*.*75 (0*.*12)

*−*0*.*30 (0*.*44)

*−*0*.*58 (0*.*13)

∗

*−*0*.*32 (0*.*42)

*−*0*.*60 (0*.*12)

∗

*−*0*.*91 (0*.*45)

*−*0*.*72 (0*.*13)

∗

*−*0*.*93 (0*.*42)

*−*0*.*73 (0*.*12)

∗

*−*0*.*30 (0*.*44)

*−*0*.*58 (0*.*13)

∗

∗

Unempl. 0*.*12 (0*.*85) *−*0*.*74 (0*.*89)∗

*−*0*.*21 (0*.*83)∗

*−*0*.*30 (0*.*91) 0*.*00 (0*.*85) *−*0*.*76 (0*.*89)∗

SDO *−*2*.*05 (0*.*14)∗

Authoritarianism *−*0*.*76 (0*.*26)∗

Ethnocentrism *−*1*.*55 (0*.*22)

Interactions

*−*1*.*83 (0*.*15)

*−*1*.*11 (0*.*25)^∗^

*−*1*.*18 (0*.*28)^∗^

*−*2*.*05 (0*.*14)

*−*0*.*41 (0*.*30)

∗

*−*1*.*55 (0*.*22)

Non-WEIRD 1*.*88 (0*.*22)^∗^ 1*.*94 (0*.*24)^∗^ 1*.*03 (0*.*57) 1*.*24 (0*.*25)^∗^ 2*.*36 (0*.*29)^∗^ 2*.*42 (0*.*31)^∗^ Non-WEIRD x SDO 0*.*74 (0*.*13)^∗^

*−*

*— − − − −*

Non-WEIRD x authorit. 0*.*63 (0*.*25)^∗^ 0*.*62 (0*.*27)^∗^

*— −*

Non-WEIRD x ethnoc. 1*.*88 (0*.*22)^∗^

*−*

Country dummies

Germany *−*2*.*09 (0*.*72)^∗^ *−*2*.*27 (0*.*77)^∗^ *−*1*.*79 (0*.*71)^∗^ *−*2*.*43 (0*.*78)^∗^ *−*2*.*32 (0*.*72)^∗^ *−*2*.*27 (0*.*77)^∗^

Denmark *−*5*.*68 (0*.*86)^∗^ *−*6*.*49 (0*.*91)^∗^ *−*6*.*39 (0*.*85)^∗^ *−*5*.*63 (0*.*92)^∗^ *−*5*.*77 (0*.*86)^∗^ *−*6*.*49 (0*.*91)^∗^

Sweden *−*4*.*82 (0*.*84)^∗^ *−*6*.*86 (0*.*92)^∗^ *−*6*.*51 (0*.*84)^∗^ *−*5*.*24 (0*.*92)^∗^ *−*4*.*77 (0*.*84)^∗^ *−*6*.*86 (0*.*92)^∗^

United Kingdom *−*14*.*44 (0*.*85)^∗^ *−*16*.*65 (0*.*93)^∗^ *−*15*.*65 (0*.*84)^∗^ *−*15*.*83 (0*.*94)^∗^ *−*14*.*29 (0*.*85)^∗^ *−*16*.*65 (0*.*93)^∗^

United States *−*9*.*35 (0*.*74)^∗^ *−*11*.*31 (0*.*81)^∗^ *−*10*.*79 (0*.*73)^∗^ *−*10*.*59 (0*.*81)^∗^ *−*8*.*69 (0*.*74)^∗^ *−*11*.*31 (0*.*81)^∗^

AIC 253913*.*03 229823*.*03 253588*.*41 229993*.*50 253873*.*15 229820*.*53

BIC 254234*.*66 230157*.*08 253926*.*54 230319*.*41 254211*.*27 230162*.*73

Log Likelihood 126917*.*51 114870*.*51 126753*.*21 114956*.*75 126895*.*58 114868*.*26

*— − − − − −*

Obervations 28195 25531 28195 25531 28195 25531

Respondents 9399 8511 9399 8511 9399 8511

Var: Resp. intercept 293*.*63 288*.*46 285*.*07 295*.*79 292*.*82 288*.*54

Var: Non-WEIRD 1*.*06 3*.*65 0*.*68 0*.*68 0*.*98 3*.*41

Cov: intercept-Non-WEIRD 17*.*66 9*.*71 13*.*94 14*.*21 16*.*96 9*.*79

Var: Residual 294*.*89 296*.*77 294*.*51 296*.*56 294*.*81 296*.*74

∗ *p <* 0*.*05

Table S-18: Interactive models – Alternative immigrant coding (WEIRD vs. Non- WEIRD; pooled)

Germany: beta = 0.19 (t = 0.13; p = 0.9) United States: beta = 0.35 (t = 0.22; p = 0.83)

0 2

1

Avg. marginal effect of being dark−skinned

Avg. marginal effect of being dark−skinned

−1

0

−2

−1

−3

−2

−0.1 0.0 0.1 0.2 0.3 0.4 0.5 0.6 0.7 0.8 0.9 1.0

IAT D−score

95% confidence intervals.

- - 1. Germany

−0.2 −0.1 0.0 0.1 0.2 0.3 0.4 0.5 0.6 0.7 0.8 0.9

IAT D−score

95% confidence intervals.

- - 1. United States

Figure S-15: Implicit bias does not have a conditioning effect: Alternative coding of immigrant attribute (light- vs. dark-skinned)

*Notes:* See Tables [S-20](#_bookmark43) and [S-19](#_bookmark42) for the detailed estimation results.

Model 1

Intercept 51*.*78 (2*.*63)^∗^

Vignette attributes

Female 1*.*42 (0*.*55)^∗^

Age: 40 years 0*.*85 (0*.*66)

Age: 55 years 1*.*18 (0*.*67)

Commute too long 5*.*91 (0*.*79)^∗^

Excessive overtime 6*.*68 (0*.*79)^∗^

Closure due to COVID 16*.*63 (0*.*78)^∗^

Tax/Contr: 2 years 0*.*93 (0*.*77)

Tax/Contr: 4 years 1*.*32 (0*.*78)

Tax/Contr: 8 years 3*.*82 (0*.*77)^∗^

Residency: 10 years 2*.*29 (0*.*77)^∗^

*−*

Residency: 5 years 2*.*37 (0*.*77)^∗^

*−*

Residency: 2 years 2*.*73 (0*.*78)^∗^

*−*

Lab technician 0*.*20 (0*.*78)

Food engineer 0*.*02 (0*.*78)

Accountant 0*.*02 (0*.*78)

*−*

Up to 3 months 1*.*25 (0*.*78)

∗

*−*

Up to 6 months 1*.*72 (0*.*78)

*−*

Up to 1 year 1*.*29 (0*.*77)

*—* ∗

Effort: Looking, 1-2 appl./week 7*.*81 (0*.*79)

Effort: Looking, 3-4 appl./week 8*.*48 (0*.*79)^∗^

Effort: Looking, 5-6 appl./week 9*.*22 (0*.*78)^∗^ Resp. controls

Resp. age 0*.*11 (0*.*03)^∗^

*−*

Resp. not DE-born 0*.*03 (2*.*37)

*−*

Upper sec./post-sec. non-tertiary 0*.*62 (1*.*29) Tertiary (BA, MA, Professional, PhD) 0*.*68 (1*.*51) Male 1*.*15 (1*.*04)

*−*

*−*

Available savings 0*.*42 (0*.*30)

*−*

Unempl. 3*.*56 (2*.*78)

Interaction

Dark-skinned 1*.*57 (0*.*90)

*−*

IAT D-score 0*.*35 (1*.*33)

Dark-skinned x IAT D-score 0*.*19 (1*.*47)

AIC 33084*.*45

BIC 33308*.*69

Log Likelihood 16506*.*23

*−*

Observations 3747

Respondents 1249

Var: Resp. intercept 225*.*80

Var: Dark-skinned 43*.*13

Cov: intercept-Dark-skinned 13*.*39

Var: Residual 244*.*37

∗*p <* 0*.*05

Table S-19: Models of effects of IAT *D*-scores (DE) – alternative immigrant at- tribute coding (light- vs. dark-skinned)

Model 1

Intercept 57*.*82 (5*.*42)^∗^

Vignette attributes

Female 1*.*03 (0*.*66)

Age: 40 years 1*.*44 (0*.*79)

Age: 55 years 2*.*36 (0*.*80)^∗^

Commute too long 4*.*54 (0*.*94)^∗^

Excessive overtime 2*.*69 (0*.*93)^∗^

Closure due to COVID 15*.*77 (0*.*92)^∗^

Tax/Contr: 2 years 1*.*08 (0*.*93)

Tax/Contr: 4 years 1*.*27 (0*.*92)

Tax/Contr: 8 years 2*.*49 (0*.*93)^∗^

Residency: 10 years 2*.*14 (0*.*93)^∗^

*−*

Residency: 5 years 2*.*68 (0*.*95)^∗^

*−*

Residency: 2 years 2*.*54 (0*.*92)^∗^

*−*

Lab technician 0*.*12 (0*.*93)

Food engineer 0*.*23 (0*.*93)

*−*

Accountant 1*.*12 (0*.*94)

*−*

Up to 3 months 0*.*64 (0*.*95)

*—* ∗

Up to 6 months 2*.*10 (0*.*93)

*—* ∗

Up to 1 year 3*.*14 (0*.*93)

*—* ∗

Effort: Looking, 1-2 appl./week 9*.*52 (0*.*93)

Effort: Looking, 3-4 appl./week 9*.*42 (0*.*95)^∗^

Effort: Looking, 5-6 appl./week 11*.*54 (0*.*93)^∗^ Resp. controls

Resp. age 0*.*12 (0*.*04)^∗^

*−*

Resp. not US-born 2*.*34 (2*.*77) Upper sec./post-sec. non-tertiary 10*.*30 (4*.*84)^∗^ Tertiary (BA, MA, Professional, PhD) 9*.*30 (4*.*87) Male 0*.*02 (1*.*30)

*—* ∗

*−*

*−*

Black/Afr.Amer. 7*.*77 (2*.*54)

Asian/Pac.Isl. 0*.*53 (3*.*20)

*—* ∗

Hispanic 8*.*49 (3*.*86)

Other 6*.*96 (3*.*78)

∗

*−*

Available savings 1*.*24 (0*.*36)

*−*

Unempl. 0*.*77 (1*.*88)

*−*

Interaction

Dark-skinned 0*.*03 (0*.*95)

IAT D-score 0*.*96 (1*.*66)

*−*

Dark-skinned x IAT D-score 0*.*35 (1*.*61)

AIC 30503*.*37

BIC 30748*.*12

Log Likelihood 15211*.*68

*−*

Observations 3357

Respondents 1119

Var: Resp. intercept 325*.*56

Var: Dark-skinned 19*.*25

Cov: intercept-Dark-skinned 6*.*00

*−*

Var: Residual 323*.*99

∗*p <* 0*.*05

Table S-20: Models of effects of IAT *D*-scores (US) – alternative immigrant at- tribute coding (light- vs. dark-skinned)

Germany: beta = −1.77 (t = −1.26; p = 0.21) United States: beta = −1.45 (t = −0.89; p = 0.37)

1

0

2

Avg. marginal effect of being non−WEIRD

Avg. marginal effect of being non−WEIRD

−1

−2

0

−3

−4

−2

−0.1 0.0 0.1 0.2 0.3 0.4 0.5 0.6 0.7 0.8 0.9 1.0

IAT D−score

95% confidence intervals.

- - - 1. Germany

−0.2 −0.1 0.0 0.1 0.2 0.3 0.4 0.5 0.6 0.7 0.8 0.9

IAT D−score

95% confidence intervals.

- - - 1. United States

Figure S-16: Implicit bias does not have a conditioning effect: Alternative coding of immigrant attribute (WEIRD vs. Non-WEIRD)

*Notes:* See Tables [S-22](#_bookmark46) and [S-21](#_bookmark45) for the detailed estimation results.

Model 1

Intercept 51*.*98 (2*.*66)^∗^

Vignette attributes

Female 1*.*37 (0*.*55)^∗^

Age: 40 years 0*.*86 (0*.*66)

Age: 55 years 1*.*26 (0*.*67)

Commute too long 5*.*91 (0*.*80)^∗^

Excessive overtime 6*.*78 (0*.*78)^∗^

Closure due to COVID 16*.*75 (0*.*78)^∗^

Tax/Contr: 2 years 1*.*03 (0*.*77)

Tax/Contr: 4 years 1*.*47 (0*.*78)

Tax/Contr: 8 years 3*.*85 (0*.*77)^∗^

Residency: 10 years 2*.*38 (0*.*77)^∗^

*−*

Residency: 5 years 2*.*36 (0*.*77)^∗^

*−*

Residency: 2 years 2*.*69 (0*.*77)^∗^

*−*

Lab technician 0*.*12 (0*.*78)

Food engineer 0*.*13 (0*.*78)

Accountant 0*.*04 (0*.*78)

*−*

Up to 3 months 1*.*33 (0*.*78)

∗

*−*

Up to 6 months 1*.*79 (0*.*77)

*−*

Up to 1 year 1*.*33 (0*.*77)

*—* ∗

Effort: Looking, 1-2 appl./week 7*.*63 (0*.*79)

Effort: Looking, 3-4 appl./week 8*.*31 (0*.*79)^∗^

Effort: Looking, 5-6 appl./week 9*.*12 (0*.*78)^∗^ Resp. controls

Resp. age 0*.*10 (0*.*03)^∗^

*−*

Resp. not DE-born 0*.*17 (2*.*38)

Upper sec./post-sec. non-tertiary 0*.*61 (1*.*29) Tertiary (BA, MA, Professional, PhD) 0*.*73 (1*.*52) Male 1*.*17 (1*.*04)

*−*

*−*

Available savings 0*.*41 (0*.*30)

*−*

Unempl. 3*.*51 (2*.*78)

Interaction

Non-WEIRD 1*.*23 (0*.*87)

*−*

IAT D-score 1*.*38 (1*.*45)

Non-WEIRD x IAT D-score *−*1*.*77 (1*.*41)

AIC 33080*.*46

BIC 33304*.*70

Log Likelihood 16504*.*23

*−*

Observations 3747

Respondents 1249

Var: Resp. intercept 216*.*38

Var: Non-WEIRD 29*.*24

Cov: intercept-Non-WEIRD 12*.*98

Var: Residual 247*.*52

∗*p <* 0*.*05

Table S-21: Models of effects of IAT *D*-scores (DE) – alternative immigrant at- tribute coding (WEIRD vs. Non-WEIRD)

Model 1

Intercept 57*.*31 (5*.*44)^∗^

Vignette attributes

Female 1*.*05 (0*.*66)

Age: 40 years 1*.*44 (0*.*79)

Age: 55 years 2*.*34 (0*.*80)^∗^

Commute too long 4*.*56 (0*.*94)^∗^

Excessive overtime 2*.*72 (0*.*93)^∗^

Closure due to COVID 15*.*78 (0*.*92)^∗^

Tax/Contr: 2 years 1*.*11 (0*.*93)

Tax/Contr: 4 years 1*.*33 (0*.*92)

Tax/Contr: 8 years 2*.*57 (0*.*93)^∗^

Residency: 10 years 2*.*12 (0*.*93)^∗^

*−*

Residency: 5 years 2*.*65 (0*.*94)^∗^

*−*

Residency: 2 years 2*.*53 (0*.*92)^∗^

*−*

Lab technician 0*.*17 (0*.*93)

Food engineer 0*.*18 (0*.*93)

*−*

Accountant 1*.*08 (0*.*94)

*−*

Up to 3 months 0*.*60 (0*.*95)

*—* ∗

Up to 6 months 2*.*04 (0*.*93)

*—* ∗

Up to 1 year 3*.*06 (0*.*93)

*—* ∗

Effort: Looking, 1-2 appl./week 9*.*56 (0*.*93)

Effort: Looking, 3-4 appl./week 9*.*43 (0*.*95)^∗^

Effort: Looking, 5-6 appl./week 11*.*61 (0*.*92)^∗^ Resp. controls

Resp. age 0*.*12 (0*.*04)^∗^

*−*

Resp. not US-born 2*.*30 (2*.*78) Upper sec./post-sec. non-tertiary 10*.*53 (4*.*84)^∗^ Tertiary (BA, MA, Professional, PhD) 9*.*50 (4*.*87) Male 0*.*03 (1*.*30)

*−*

*−*

Black/Afr.Amer. 7*.*68 (2*.*54)^∗^

Asian/Pac.Isl. 0*.*43 (3*.*20)

*—* ∗

Hispanic 8*.*42 (3*.*86)

Other 7*.*12 (3*.*78)

∗

*−*

Available savings 1*.*24 (0*.*36)

*−*

Unempl. 0*.*74 (1*.*88)

*−*

Interaction

Non-WEIRD 0*.*87 (0*.*96)

IAT D-score 0*.*08 (1*.*84)

Non-WEIRD x IAT D-score *−*1*.*45 (1*.*63)

AIC 30502*.*40

BIC 30747*.*15

Log Likelihood 15211*.*20

*−*

Observations 3357

Respondents 1119

Var: Resp. intercept 337*.*39

Var: Non-WEIRD 7*.*84

Cov: intercept-Non-WEIRD 14*.*13

*−*

Var: Residual 327*.*33

∗*p <* 0*.*05

Table S-22: Models of effects of IAT *D*-scores (US) – alternative immigrant at- tribute coding (WEIRD vs. Non-WEIRD)

# References

Crepaz, Markus ML. 2020. “Coveting Uniformity in a Diverse World: The Author- itarian Roots of Welfare Chauvinism in Postmigration Crisis Germany.” *Social Science Quarterly* 101(4):1255–1270.

Kam, Cindy D. and Donald R. Kinder. 2012. “Ethnocentrism as a short-term force in the 2008 American presidential election.” *American Journal of Political Science* 56(2):326–340.

Nosek, Brian A., Frederick L. Smyth, Jeffrey J. Hansen, Thierry Devos, Nicole M. Lindner, Kate A. Ranganath, Colin Tucker Smith, Kristina R. Olson, Dolly Chugh, Anthony G. Greenwald and Mahzarin R. Banaji. 2007. “Pervasiveness and correlates of implicit attitudes and stereotypes.” *European Review of Social Psychology* 18(1):36–88.

Pratto, Felicia, Jim Sidanius, Lisa M. Stallworth and Bertram F. Malle. 1994. “So- cial dominance orientation: A personality variable predicting social and political attitudes.” *Journal of Personality and Social Psychology* 67(4):741–763.

Tillman, Erik R. 2013. “Authoritarianism and citizen attitudes towards European integration.” *European Union Politics* 14(4):566–589.
